# The High Energy Density Scientific Instrument at the European XFEL

**DOI:** 10.1107/S1600577521007335

**Published:** 2021-08-23

**Authors:** Ulf Zastrau, Karen Appel, Carsten Baehtz, Oliver Baehr, Lewis Batchelor, Andreas Berghäuser, Mohammadreza Banjafar, Erik Brambrink, Valerio Cerantola, Thomas E. Cowan, Horst Damker, Steffen Dietrich, Samuele Di Dio Cafiso, Jörn Dreyer, Hans-Olaf Engel, Thomas Feldmann, Stefan Findeisen, Manon Foese, Daniel Fulla-Marsa, Sebastian Göde, Mohammed Hassan, Jens Hauser, Thomas Herrmannsdörfer, Hauke Höppner, Johannes Kaa, Peter Kaever, Klaus Knöfel, Zuzana Konôpková, Alejandro Laso García, Hanns-Peter Liermann, Jona Mainberger, Mikako Makita, Eike-Christian Martens, Emma E. McBride, Dominik Möller, Motoaki Nakatsutsumi, Alexander Pelka, Christian Plueckthun, Clemens Prescher, Thomas R. Preston, Michael Röper, Andreas Schmidt, Wolfgang Seidel, Jan-Patrick Schwinkendorf, Markus O. Schoelmerich, Ulrich Schramm, Andreas Schropp, Cornelius Strohm, Konstantin Sukharnikov, Peter Talkovski, Ian Thorpe, Monika Toncian, Toma Toncian, Lennart Wollenweber, Shingo Yamamoto, Thomas Tschentscher

**Affiliations:** a European XFEL, Holzkoppel 4, 22869 Schenefeld, Germany; b Helmholtz-Zentrum Dresden-Rossendorf eV, 01328 Dresden, Germany; c Deutsches Elektronen-Synchrotron DESY, 22607 Hamburg, Germany; e Technische Universität Dortmund, 44227 Dortmund, Germany; f SLAC National Accelerator Laboratory, Menlo Park, CA 94025, USA

**Keywords:** high energy density, X-ray free-electron lasers, warm dense matter, high-pressure science, relativistic laser–matter interaction

## Abstract

The European XFEL delivers up to 27000 intense (>10^12^ photons) pulses per second, of ultrashort (≤50 fs) and transversely coherent X-ray radiation, at a maximum repetition rate of 4.5 MHz. Its unique X-ray beam parameters enable groundbreaking experiments in matter at extreme conditions at the High Energy Density (HED) scientific instrument. The performance of the HED instrument during its first two years of operation, its scientific remit, as well as ongoing installations towards full operation are presented. Scientific goals of HED include the investigation of extreme states of matter created by intense laser pulses, diamond anvil cells, or pulsed magnets, and ultrafast X-ray methods that allow their diagnosis using self-amplified spontaneous emission between 5 and 25 keV, coupled with X-ray monochromators and optional seeded beam operation. The HED instrument provides two target chambers, X-ray spectrometers for emission and scattering, X-ray detectors, and a timing tool to correct for residual timing jitter between laser and X-ray pulses.

## Abstract 2

The unique parameters of the European XFEL enable novel and groundbreaking experiments in matter at extreme conditions at the High Energy Density (HED) scientific instrument. The performance of the HED instrument during its first two years of operation, its scientific remit, as well as ongoing installations towards full operation are presented.

## 1. Scientific scope   

The High Energy Density (HED) scientific instrument at the European X-ray Free-Electron Laser Facility GmbH (European XFEL) (Tschentscher *et al.*, 2017 ▸; Decking *et al.*, 2020 ▸) is a unique platform for experiments combining hard X-ray free-electron laser radiation with the capability to generate matter at extreme conditions of pressure, temperature, or electromagnetic fields. The instrument started user operation in May 2019 and will augment its capabilities within the next years.

The scientific aims of the HED instrument are summarized in the HED Conceptual Design Report (CDR) (Nakatsutsumi & Tschentscher, 2013 ▸). In general, HED science sheds light on properties of matter with an energy density exceeding 10^11^ J m^−3^, which is equivalent to a pressure of >1 Mbar, a magnetic field strength of 500 T, or the internal energy of a hydrogen atom (Drake, 2010 ▸). These conditions prevail, for example, in planetary and stellar interiors, impact scenarios, and in intense laser–matter interactions in both fundamental research and industrial applications.

Located downstream of the SASE2 undulator, which provides photon energies of 5–25 keV, the HED instrument focuses on experiments which require hard X-rays, allowing volumetric studies of high-density material, pumping certain atomic resonances or covering a large reciprocal space in crystallographic structure analysis. In particular, the instrument is optimized to exploit the pulse structure of the European XFEL, offering an up to 600 µs-long train of X-ray pulses each separated by 222 ns [equivalent to a rate of (222 ns)^−1^ = 4.514 MHz], at a base repetition rate of 10 Hz, therefore delivering a bright X-ray burst every 100 ms. Single or multiple pulses in a train at 10 Hz or on demand can be selected. The provided instrumentation is tailored to match this 10 Hz repetition rate and to make use of the unique pulse train structure. In comparison with conventional HED platforms, this increases the data rate and hence leads to a paradigm shift regarding statistics and the ability to explore a wider parameter space during a single experimental campaign. The unique MHz capability means that the evolution of sub-millisecond dynamics can be recorded with a single X-ray burst. This is particularly interesting for processes which do not permit accumulative measurements or cannot be repeated frequently (Cerantola *et al.*, 2021 ▸). Key scientific cases for the HED instrument are summarized below.

At gigapascal pressures, complex crystallographic and chemical states of matter may form (Appel *et al.*, 2014 ▸). At the HED instrument, static high-pressure conditions of several 100 GPa can be generated using diamond anvil cells (DACs). The pressure can be dynamically varied using DACs equipped with piezoactuators (dynamic DAC, dDAC) (Jenei *et al.*, 2019 ▸) and the temperatures increased via absorption of X-rays (Meza-Galvez *et al.*, 2020 ▸) or infrared lasers (Konôpková *et al.*, 2016 ▸).

Studying the response of materials to different strain rates allows to differentiate between plastic and elastic processes and study the kinetics of phase transitions. Dynamic loading at even higher strain rates and to higher pressures can be studied in shock wave experiments using laser ablation pressure. A diode-pumped laser (De Vido *et al.*, 2019 ▸) with 2–15 ns pulse duration at 10 Hz (Section 5.3.2) providing up to 100 J in the infrared will enable the generation of transient pressures of up to 1 TPa (Zastrau *et al.*, 2017 ▸). Such ultrahigh pressures lead to discoveries of unique physical and chemical phenomena and a deeper understanding of matter.

A combination of a femtosecond (fs) duration, high-intensity optical laser with an XFEL allows to study transient, non-equilibrium states on ultrafast time scales (Nakatsutsumi *et al.*, 2016 ▸). This allows the study of the electron–ion thermalization dynamics, non-thermal melting and associated transport properties under *a priori* known ion density, to name a few. Understanding these processes provides deeper insight into material processing via ablation and warm dense matter (WDM) – a dense plasma state between the ideal solid and an ideal plasma. At electron-relativistic laser intensity (≥10^18^ W cm^−2^), generation of the extremely high magnetic fields (Wang *et al.*, 2019 ▸), growth rates of instabilities (Ruyer *et al.*, 2020 ▸; Göde *et al.*, 2017 ▸) and collisionless shocks (Fiuza *et al.*, 2020 ▸) can be investigated. Fundamental understanding of these transient phenomena also helps to optimize and control the generation of bright particles (Wilks *et al.*, 2001 ▸), X-ray line emission (Zastrau *et al.*, 2010 ▸) and coherent attosecond extreme ultraviolet pulses (Wheeler *et al.*, 2012 ▸) as a secondary source for a probe or a driver.

Instead of using an optical laser which interacts with only the sample surface, the volumetric formation of WDM using intense X-rays has shown great potential over the last decade (Vinko *et al.*, 2012 ▸; Sperling *et al.*, 2015 ▸.). Studies can be readily extended to X-ray pump/X-ray probe techniques using a split-and-delay line (Lu *et al.*, 2018 ▸), as well as two-color operation of the XFEL itself.

A pulsed magnetic field setup allows the stabilization of otherwise inaccessible new states of matter. Of interest are correlated electron systems where spin, orbital, charge and lattice degrees of freedom act on similar energy scales and lead to competing ground states. One of these so far unknown phases is the state of high-*T*_c_ superconductors above the critical magnetic field, when the superconducting property has collapsed (Grissonnanche *et al.*, 2016 ▸). Other systems of current interest are frustrated magnets from triangular lattices that show rich phase diagrams at high magnetic fields (Wosnitza *et al.*, 2016 ▸), and materials showing topological order (Liu *et al.*, 2017 ▸).

## 2. The HIBEF user consortium   

The Helmholtz International Beamline for Extreme Fields (HIBEF) user consortium (UC) contributes instrumentation and operation staff to the HED instrument. HIBEF is led by the Helmholtz Zentrum Dresden-Rossendorf (HZDR), and other major contributors are the Deutsches Elektronen-Synchrotron (DESY) and the UK’s Scientific and Technology Facilities Council (STFC). The HIBEF-provided instrumentation represents an integral part of the HED instrument – major contributions of the UC include high-intensity and high-energy laser systems (see Sections 5.3.1 and 5.3.2), a dedicated interaction chamber for precision X-ray diffraction (XRD) studies (see Section 6.4) in laser-shock and DAC experiments, and a setup for studying samples at cryogenic temperatures in pulsed magnetic fields (see Sections 5.4 and 6.5). While the European XFEL is responsible for the provision of beam time, staff from HIBEF and the European XFEL operate, maintain and develop the experimental platforms jointly. European XFEL publishes an open call for proposals typically twice a year in May and November.

## 3. Technical infrastructure   

### 3.1. European XFEL and the SASE2 undulator source   

**Figure 1:**
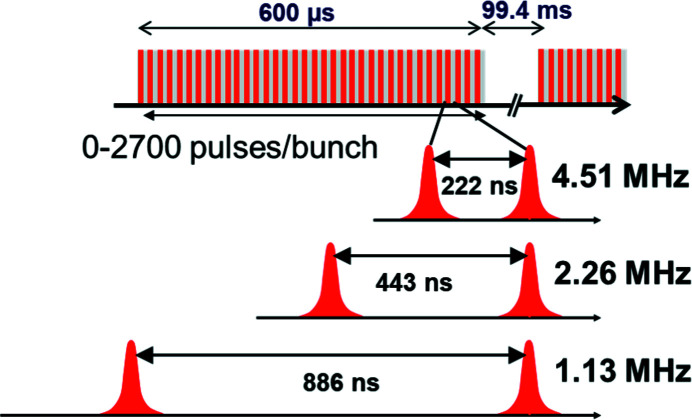
Unique bunch pattern of European XFEL. Within an up to 600 µs-long window, between a single and 2700 X-ray pulses can be created. The facility can deliver pulses at difference repetition rates of 4.51, 2.26, or 1.13 MHz, resulting in a pulse spacing of 222, 443 or 886 ns, respectively.

The European XFEL is a free-electron laser (FEL) user facility currently delivering soft and hard X-ray FEL radiation to six scientific instruments (Tschentscher *et al.*, 2017 ▸). It provides X-rays with high average brilliance and extreme peak intensities at photon energies up to 25 keV in the fundamental, with femtosecond pulse duration and a high degree of coherence. The high average brilliance is achieved through acceleration of up to 2700 electron bunches with typically 0.25 nC at a 10 Hz repetition rate by a superconducting electron accelerator (Weise & Decking, 2017 ▸) (Fig. 1 ▸). After reaching energies of 11.5, 14, or 16.5 GeV, these electrons are distributed to three beamlines housing self-amplified spontaneous emission (SASE) undulators (Abeghyan *et al.*, 2019 ▸) with horizontal polarization. Tuning of the photon energy is achieved independently in each beamline by adjusting the undulator gaps. The HED science instrument is located after the SASE2 undulator which produces short ∼25 fs X-ray pulses with photon energies from 5 to 25 keV (Scholz & Zhao, 2019 ▸).

**Figure 2:**
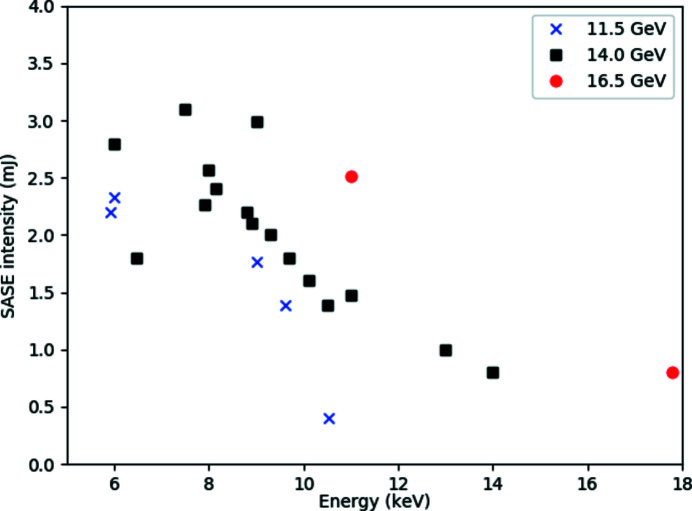
Current peak performance of the SASE2 FEL at photon energies between 6 and 18 keV. The data points represent peak pulse energies measured in 2019 and 2020 (https://xfel.desy.de/operation/performance/; see also Maltezopoulos *et al.*, 2019 ▸). The linac was operated at 11.5 GeV (blue crosses), 14 GeV (black rectangles) and 16.5 GeV (red circles) electron energy.

The highest achieved SASE2 levels are summarized in Fig. 2 ▸ – average performance is at 50–80% of these values. Typically, a SASE spectrum has a fully tunable Gaussian spectral envelope with bandwidth Δ*E*/*E* ≃ 10^−3^. Within this bandwidth, the spectrum consists of several longitudinal modes leading to <1 eV broad spikes which statistically fluctuate from pulse to pulse. Example spectra are shown in Section 4.2.2. SASE2 can also operate in hard X-ray self-seeding mode (HXRSS) (Geloni *et al.*, 2011 ▸, 2019 ▸; Liu *et al.*, 2019 ▸), producing a more coherent and narrow-bandwidth pulse. Current studies performed at 7.5 and 9 keV photon energy yield pulse energies of ∼1 mJ in a spectral bandwidth of ∼1 eV.

The initial beam divergence from the undulator can be measured using imagers along the beam transport, which in turn allows to estimate the diffraction-limited source size as a function of photon energy, as shown in Table 1 ▸.

### 3.2. HED instrument layout   

**Figure 3:**
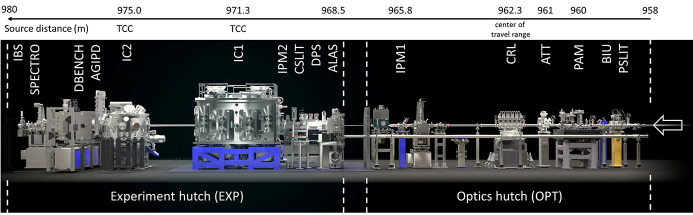
HED optics and experiment hutch layout. The X-rays enter from the right. Optics hutch: PSLIT – water-cooled four-blade power slits, BIU – beam imaging unit, PAM – photon arrival monitor for X-ray-optical laser timing, ATT – solid attenuator foils, CRL – compound refractive lenses made of Be (CRL3), IPM1 – intensity and position monitor. Experimental hutch: ALAS: incoupling for alignment laser, DPS – differential pumping, CSLIT – clean-up slits, two four-blade assemblies for soft and hard X-rays, respectively, IPM2 – intensity and position monitor, IC1 and IC2 – interaction chambers, AGIPD – MHz repetition compatible X-ray detector, DBENCH – detector bench, SPECTRO – position of downstream spectrometers, IBS – instrument beam stop. The distances on the top are given from the source (center of last undulator segment).

The HED instrument is located in the experiment hall at the end of a ∼970 m-long photon beam transport tunnel partially shared with the Materials and Imaging Dynamics (MID) instrument (Madsen *et al.*, 2021 ▸). The basic layout of the HED hutches is described in the Technical Design Report (TDR) (Nakatsutsumi *et al.*, 2014 ▸) and the layout of the optics and experiment hutch is shown in Fig. 3 ▸.

**Figure 4:**
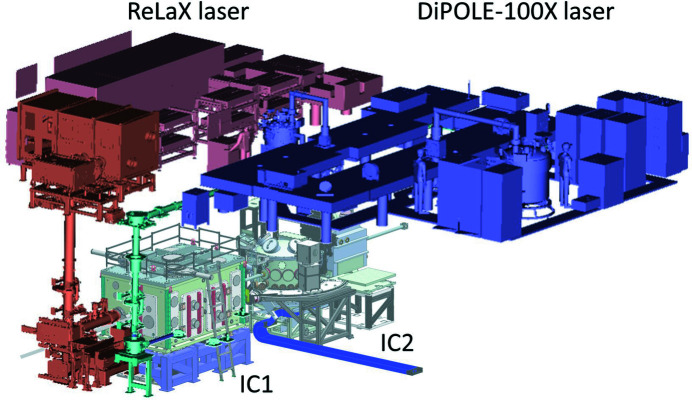
Schematics showing the position of the ReLaX and DiPOLE 100-X laser in a room above the experiment hutch with the interaction chambers IC1,2 (walls not shown).

The experiment hutch and the optics hutch are air-conditioned and H14-filtered and kept at a temperature of 21 ± 1°C and a humidity of 50 ± 2.5%. Dedicated zones exist with precision temperature control: 21 ± 0.1°C. Details of the experiment hutch arrangement are described in Section 6. On the roof of the experiment hutch is a laser bay which houses the two large laser systems ReLaX and DiPOLE (see Fig. 4 ▸ and Sections 5.3.1 and 5.3.2) in a clean-room-like laboratory with precision temperature (21 ± 0.1°C) control. Adjacent to this are rack rooms where the majority of the control electronics and power supplies are placed.

### 3.3. Control systems   

The European XFEL’s Python-based software framework for control and data acquisition is named *Karabo* (Heisen *et al.*, 2013 ▸). It allows the user to build extendable distributed applications that can be remotely controlled through a graphical user interface (GUI) or command line interface. While acting as a control system, *Karabo* interfaces with hardware controllers via driver devices. The communication between the *Karabo* devices as well as instrument- and experiment-specific programming can be implemented in so-called *Karabo* middle layer devices and macros (Hauf *et al.*, 2019 ▸).

The control hardware can be divided into two categories: slow real-time control (motion control, vacuum system, water flow and temperature control) and fast electronics for timing and analog-digital conversion. The slow real-time control is managed by an industrial standard Programmable Logic Controller (PLC) system based on EtherCAT bus terminals and CPUs from Beckhoff (Gessler *et al.*, 2020 ▸) enhanced by multi-channel-Piezo motion controllers with interlock capability that are directly interfaced to the Karabo control system.

The use of the industrial PLC system facilitates standardization and modularization of software and hardware. It also allows the implementation of equipment protection interlocks, motorized axis coupling, and feedback control. The fast electronics platform uses MicroTCA crates for timing, digitization of analog signals, and online signal processing (Gessler *et al.*, 2020 ▸) which matches the pulse rate of European XFEL.

The timing precision performance of the *Karabo*-controlled software layer is typically well below the 100 ms, but high network traffic can cause latency. Therefore, for 10 Hz pulse-to-pulse actions such as triggering an acquisition, the real-time PLCs are preferred. Sampling of data within the pulse train with 222 ns intervals, or even resolving sub-ns pulse shapes, is possible using the the MicroTCA with sampling rates of up to 0.5 Gigasamples s^−1^.

## 4. X-ray transport, optics and diagnostics   

**Figure 5:**
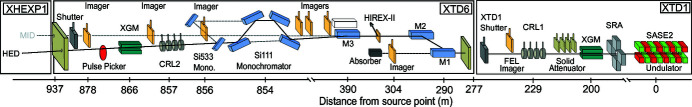
Beam transport in the XTD1 and XTD6 underground tunnels up to the XFEL Headquarters EXPeriment hall 1 (XHEXP1). The X-rays enter from the right. Not all components are shown. A detailed list of devices and distances can be found in Appendix *B* [App appb].

A schematic overview of the ∼1 km-long X-ray beam transport from the SASE2 undulator to the HED instrument beam stop is given in Fig. 5 ▸. For the first 390 m, the HED and MID instruments share a common beam transport. The first optics are a set of interchangeable lenses (CRL1) that allow the beam to be collimated or focused directly to the sample position (Nakatsutsumi & Tschentscher, 2013 ▸; Nakatsutsumi *et al.*, 2014 ▸). At photon energies of 6 keV, the beam size at CRL1 is just below 1 mm full width at half-maximum (FWHM) in accordance with design parameters (Schneidmiller & Yurkov, 2011 ▸). Next comes a pair of horizontal deflecting mirrors (M1, M2), with a usable length of 80 cm, which horizontally offset the beam in order to suppress high harmonics as well as bremsstrahlung and spontaneous synchrotron radiation.

Higher harmonics are weak multiples of the fundamental photon energy. While odd harmonics are created in every undulator on-axis, even ones are found in a cone slightly off-axis. With chromatic focusing optics (such as beryllium lenses) they are not focused to the same spot as the fundamental. In particular, the strong third harmonic often disturbs diffraction patters and creates X-ray fluorescence. On the other hand, it can be used as hard X-ray source in excess of the 25 keV limit of the fundamental.

**Figure 6:**
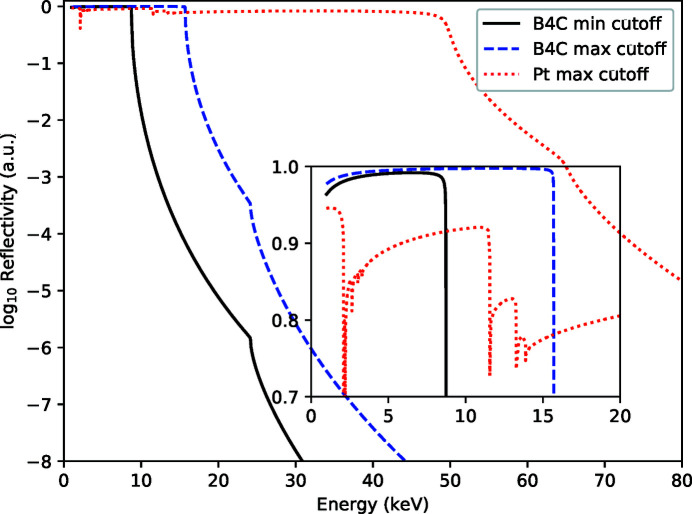
Calculated total external reflectivity of the combined mirrors M1–M3 for both reflecting coatings plotted on a log scale, inset on linear scale. Shown is the minimum energy cut-off using the B_4_C coating for all three mirrors and mirrors M1 and M2 at 3.6 mrad suitable to reduce third harmonic content, and the maximum energy cut-off using the Pt coating for all three mirrors and mirrors M1 and M2 at 1.7 mrad. Also shown is the typically used working point at 2 mrad using the B_4_C coating. In all cases the mirror M3 is set to its working point of 1.3 mrad.

The grazing-incidence angle of M1 and M2 can be varied between 1.7 and 3.6 mrad and the mirrors have both B_4_C and Pt coatings for low and high photon energy operation, respectively, to ensure high reflectivity across the entire photon energy range. The reflectivity is shown in Fig. 6 ▸. A third, mechanically identical mirror (M3) can be inserted at 390 m with a fixed incidence angle of 1.3 mrad to steer the beam to the HED instrument (Tschentscher *et al.*, 2017 ▸; Sinn *et al.*, 2019 ▸).

Even in the case of a collimated beam, overfilling of the M3 mirror can occur at mid-to-low photon energy (≤15 keV) (Nakatsutsumi & Tschentscher, 2013 ▸). To overcome this problem, an intermediate focusing scheme before the downstream optics using the CRL1 can be applied. In addition, M2 is bendable which allows both the correction of the horizontal beam size and potential astigmatism from thermal mirror deformation (Vannoni *et al.*, 2019 ▸). The transmission of the beamline over the mirrors is typically ∼70–80%.

The vertical divergence of the X-ray beam can be adjusted to match horizontal divergence by bending the M2 mirror. The resulting beam has a circular shape; however, beam jitter and weak diffraction from the M1–M3 mirror edges result in a slight horizontally broadening.

Changes in the beam pointing from the undulator, or thermal drift of mirrors M1–M3, can lead to large beam drifts after ∼1 km of photon beam transport. Therefore an active beam stabilization system has been implemented. The X-ray beam pointing is constantly monitored by highly transmissive boron-doped chemical vapor deposition (CVD) diamond screens downstream of the mirrors. A feedback loop acts on piezo actuators on mirrors M2 and M3 to correct the beam pointing.

### 4.1. X-ray focusing optics   

The X-ray focusing is based on Be CRLs (Lengeler *et al.*, 1999 ▸). These optics are chromatic, which requires different lens configurations for each photon energy (Zozulya *et al.*, 2019 ▸; Nakatsutsumi *et al.*, 2014 ▸).

Three groups of lenses are placed at different positions along the HED beamline (labeled CRL1-3) in order to ensure the maximum throughput of photons by matching the aperture to the FEL beam size. Several focal schemes and the lens configurations are shown in Appendix *B* [App appb]. Depending on photon energy and not taking into account the chromatic aberration due to the finite SASE bandwidth, the smallest FWHM beam sizes that can be theoretically achieved at the target chamber center (TCC) are 150–250 µm with CRL1, ∼15 µm with CRL2 and 1–2 µm with CRL3. The CRL2 is placed after the monochromators in order to keep the divergence over the monochromators low.

Since the lenses are chromatic, a SASE spectrum will be focused across a finite range. Table 2 ▸ compares the Rayleigh length of the monochromatic beam with the actual focal length spread according to 0.3% FWHM spectral bandwidth using an intermediate focus with CRL1 and subsequent tight focusing with CRL3. The entire chamber of CRL3 can be translated by ±50 cm along the beam axis to overlap focal positions and TCC.

**Figure 7:**
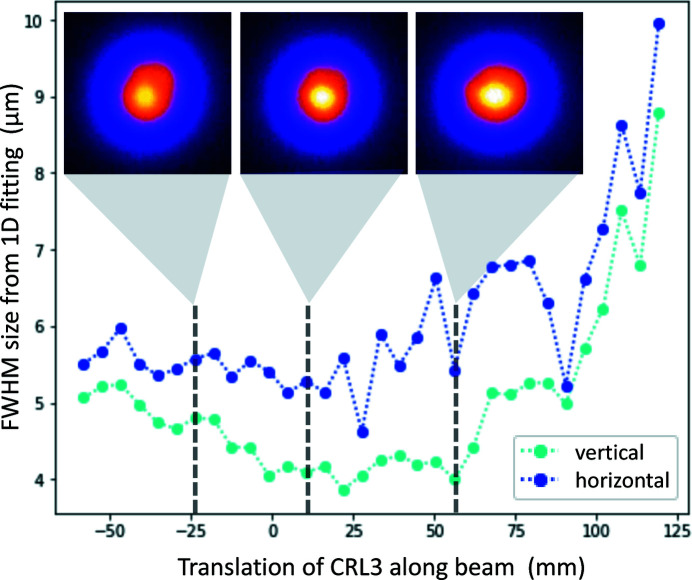
Characterization of the best focus of CRL3 and an intermediate focus scheme with CRL1 at 6 keV photon energy. The three false-color insets are single-shot imprints into a LiF crystal at 0.5% beamline transmission.

In order to characterize the shape and size of the X-ray focus, single-pulse imprints in lithium fluoride (LiF) creating permanent point-defects (Pikuz *et al.*, 2015 ▸) were taken and yield a focal spot of 4–5 µm FWHM at 6 keV with SASE beam (Fig. 7 ▸). Due to chromatic aberration, the Rayleigh range extends over several tens of mm.

**Figure 8:**
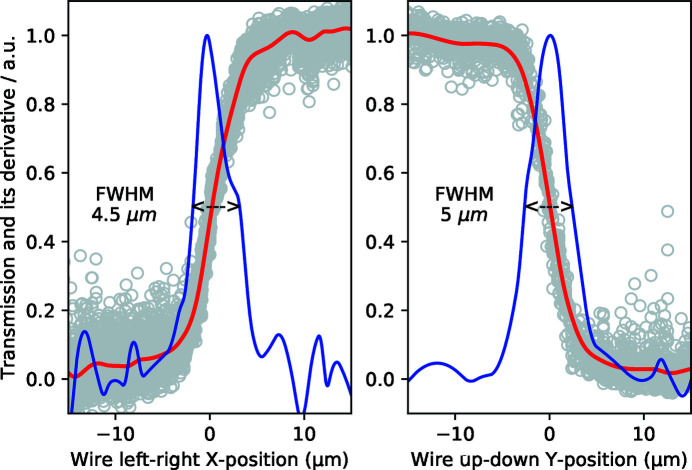
Characterization of the best focus of CRL3 after intermediate focus with CRL1 at 17.8 keV with a wire scan. The scans took a few minutes with 5% transmission. The *y*-axis shows in red the transmission (normalized to the incident pulse energy, and with a moving average), as well as its derivative in blue.

Alternatively, scanning methods with an obstacle project the focal size along the scan direction. Figure 8 ▸ shows the results of scanning the round edge of a 1 mm-diameter tungsten wire across the focused X-rays at 17.8 keV, resulting in a 4.5 µm × 5 µm spot (FWHM). While providing immediate focal size estimates, the result is potentially broadened by beam pointing jitter.

In order to reach X-ray foci smaller than 1 µm, both interaction chambers can be equipped with CRLs with adjustable short-focal length between about 10 cm and 1 m. This nano-focusing capability (CRL4) is contributed by the HIBEF UC. These lenses have radii of curvature of 50 µm, an effective aperture between 300 µm and 400 µm, and up to 50 of them can be hosted in a lens cassette, similar to the concept described by Schropp *et al.* (2012 ▸). Slight pre-focusing is necessary to match the aperture and achieve maximum throughput.

A focus spot size of ∼220 nm at the TCC of IC1 has been demonstrated using a SASE beam at 9 keV photon energy. The average focus size was retrieved from scanning ptychography (Schropp *et al.*, 2013 ▸) and yields an upper limit of 220 nm FWHM for a monochromatic beam and ≤300 nm FWHM for SASE. For the SASE case, we could not resolve a difference between a ten-shot average and a single exposure, indicating a focal jitter of less than ∼500 nm.

### 4.2. X-ray monitors and diagnostics   

#### 4.2.1. 2D imagers   

Several 2D beam imaging monitors are installed throughout the optical path. The monitors are based on fluorescent screens such as boron-doped diamond or single-crystal cerium-doped yttrium aluminium garnet (YAG:Ce) that can be inserted at an angle to the XFEL beam (Koch *et al.*, 2019 ▸; Grünert *et al.*, 2019 ▸). The visible-light fluorescence of the beam is then recorded on a 10 Hz frame-rate camera that is positioned at an angle of 90° to the X-ray beam. All imagers and their properties are listed in Appendix *B* [App appb]. Since not all imagers are permanently inserted into the beam path, recording of their data to the data acquisition system has to be actively enabled for each imager.

#### 4.2.2. Bent crystal spectrometers   

The stochastic nature of the SASE process results in spectral fluctuations from pulse to pulse. In order to record a single pulse-resolved spectrum, the use of cylindrically bent Si crystals has been practiced widely (Zhu *et al.*, 2012 ▸) as a transmissive Bragg spectrometer, albeit its application is limited to a few tens of pulses at MHz repetition rates. A recent development using cylindrically bent diamond crystals has shown comparable spectral resolution (Boesenberg *et al.*, 2017 ▸; Samoylova *et al.*, 2019 ▸) and radiation tolerance for full 4.5 MHz pulse trains at 9 keV photon energy. There are three opportunities for inserting such spectrometers into the beam path, all of them equipped with both diamond and Si crystals with various bending radii and cuts.

**Figure 9:**
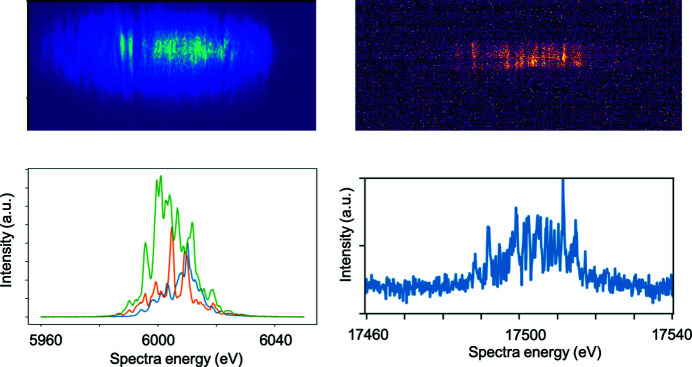
Examples of single-pulse spectra. Spectra as recorded by a 2D detector imaging a scintillator screen (top) and their corresponding lineouts (bottom). Left: HED-flex at 6 keV using a Si(111) crystal and lineouts of three different pulses to illustrate the shot-to-shot fluctuation. Right: HIREX-II in the XTD6 tunnel at 17.5 keV using C(110).

Upstream of the sample, the HIREX-II (Kujala *et al.*, 2020 ▸) spectrometer is permanently installed between mirrors M2 and M3. It covers the photon energy range 5–25 keV at a resolution of ≤0.2 eV. The reflected signal can either be recorded with a 10 Hz 2D sCMOS detector, or at 4.5 MHz with a 1D X-ray GOTTHARD detector (Mozzanica *et al.*, 2012 ▸). For experiments that require the spectral information right up- or downstream of a sample, additional bent-crystal spectrometers HED-flex and CNRS-spec can be placed simultaneously. The CNRS-spec [contribution from Center National de la Recherche Scientifique (CNRS), France] can flexibly be placed inside or outside the IC1 chamber, while the HED-flex can be placed in-air downstream of IC1. Both the HED- and CNRS-spec are designed to be inserted directly in the beam, similar to the HIREX-II spectrometer. Both are also coupled with either a 10 Hz 2D optical camera or a 1D GOTTHARD X-ray detector. The HED-flex spectrometer has been tested at 6 keV in the diverging beam (see Fig. 9 ▸) using Si(111) crystal with 78 mm bending radius, and a 10 Hz ANDOR sCMOS ZYLA 5.5 camera, imaging a 25 µm YAG:Ce screen, with a magnifying microscope objective. The minimum energy resolution for this arrangement was 0.13 eV.

A detailed description of the spectrometer configurations can be found in Appendix *B* [App appb].

#### 4.2.3. Intensity and position monitors   

X-ray gas monitors (XGMs) for non-invasive single-shot pulse energy measurements and average beam postion monitoring are installed at two positions in the HED beamline (Grünert *et al.*, 2019 ▸; Maltezopoulos *et al.*, 2019 ▸; Sorokin *et al.*, 2019 ▸). The first is placed in the XTD1 tunnel, upstream of all optics and attenuators to measure the output of the undulator. The second XGM is installed in the HED branch in the XTD6 tunnel after all the main optics. Details on their operation are described by Maltezopoulos *et al.* (2019 ▸).

Two compact intensity and position monitors (IPMs) are installed at the HED instrument. The IPM in the HED optics hutch is positioned downstream of the high-power slit system, solid attenuators and CRL3 and therefore allows the transmission of these devices to be measured. The second IPM in the experiment hutch is installed downstream of the differential pumping system and the clean-up slits. Both devices insert a thin diamond screen into the beam path and record the backscattering on four diodes (Hamamatsu S3590-09), providing pulse-resolved intensities and positions at 4.5 MHz. The signal difference between the up–down and left–right diode pairs yields the beam position. For absolute intensity measurements, the IPMs require cross-calibration to the XGMs for each specific photon energy. Since the intensity signal depends on the thickness of the screen, they have less than 2% thickness variation across the central 5 mm, and less than 5% over the entire area. Several thicknesses of CVD diamond screens are available; currently these are 22 µm, 50 µm and 100 µm. Furthermore, the instrument beam stop (IBS) also incorporates a fast diode monitoring the X-ray scattering from a permanently installed 500 µm-thick CVD diamond screen.

### 4.3. Monochromators   

**Figure 10:**
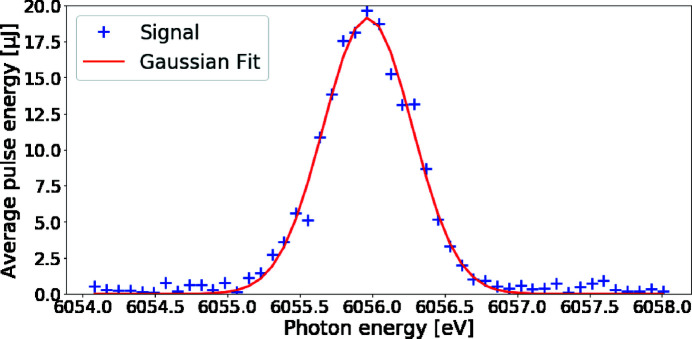
Measured energy resolution of the Si(111) monochromator at *E* = 6055 eV. The transmitted SASE X-ray pulse energy of the four-bounce setup is shown as a function of the Bragg angle (scaled to photon energy) of the second pair of crystals while the first pair was fixed. The Gaussian fit yields a FWHM of Δ*E* = 0.72 eV and Δ*E*/*E* ≃ 1.2 × 10^−4^.

An Si(111) four-bounce monochromator (Dong *et al.*, 2016 ▸) consisting of two pairs of artificial channel-cut crystals can be inserted into the beam path. It reduces the bandwidth to Δ*E*/*E* ≃ 1 × 10^−4^ (Fig. 10 ▸) for the complete range of photon energies. Both pairs of crystals can be cryogenically cooled to mitigate the heat load during a pulse train. The second pair of the Si(111)-monochromator can be replaced with a Si(533) channel-cut crystal, yielding a bandwidth of Δ*E*/*E* ≃ 4 × 10^−6^ at a fixed photon energy of 7494 eV. More details are given by Wollenweber *et al.* (2021 ▸). Both configurations provide zero offset between the incident and the monochromated beam. In order to keep the beam divergence on the crystals small, the monochromators are positioned upstream of the CRL2 and CRL3 optics. The beam jitter after the cryogenically cooled Si(111) monochromator was measured by the imager screen at the end of the XTD6 tunnel, 22 m downstream of the monochromator, to be ∼40 µm in both horizontal and vertical.

### 4.4. Slit systems   

The HED instrument includes three sets of slit systems to control the size of the beam and its potential halo. Such a halo can be caused by scattering from upstream slits, apertures, screens or filters. The water-cooled power slits are the first device located in the optics hutch, ∼15 m upstream of the TCC in IC1, and this system comprises four independent, non-intersecting slits with travel ranges of ±25 mm. The slits are 5 mm blades of tungsten carbide bonded to an absorbing block of 70 mm-thick B_4_C. The blades have highly polished knife-edges with a slope of 0.5° and can be positioned with an accuracy of 0.4 µm.

In the experiment hutch ∼2 m before the TCC of IC1, there are two sets of clean-up slits, consisting of non-intersecting blades with travel ranges of ±15 mm. These can clean up potential scattering introduced by the first slit system and the CRLs. The first pair is similar to the power slit: it uses 4 mm tungsten carbide blades, with a highly polished 0.5° knife-edge, mounted to 6 mm-thick B_4_C absorbers. The second pair, however, provides a round edge made from Si_3_N_4_ and mounted to a 3 mm tantalum blade. These slits can also be positioned with an accuracy of <1 µm.

The clean-up slits can also be used to decrease the content of higher harmonics in the beam, which is typically less focused near the TCC due to the chromaticity of the CRLs.

### 4.5. Attenuators and pulse picker   

Solid attenuators are placed in the HED beamline at two positions. The upstream attenuator is positioned in the common SASE2 branch, downstream of the first XGM and upstream of the beamline optics (see Fig. 5 ▸). It comprises six chemical vapor deposition (CVD) diamonds (0.075–2.4 mm thick) and three Si filters (0.5–2.0 mm thick).

The second attenuator at HED is installed just upstream of the CRL3 optics in the HED optics hutch. Here the filters are mounted on four motorized arms which hold six attenuators each. These 24 filters are either Si or CVD diamond with varying thicknesses (0.025 mm–6.4 mm for Si and 0.1 mm–1.6 mm for CVD diamond). A wide range of transmissions can be achieved by the insertion of filters in up to four arms simultaneously.

The pulse picker unit (PPU) can pick pulse trains at a maximum repetition rate of 10 Hz. It is positioned just downstream of the HED XGM and consists of a rotating chopper disk made from a sandwich of 2 mm B_4_C and 3 mm Densimet with several openings across its circumference. This disk is rotated by a fast DC motor.

## 5. Drivers   

### 5.1. Intense X-ray pulses   

Assuming a typical performance of the SASE2 undulator at 6–10 keV of 2 mJ pulse energy (reduced to ∼1 mJ at the TCC due to the overall beamline transmission) in a duration of 25 fs allows to create power densities, or peak intensities, in excess of 10^17^ W cm^−2^ when focused to 5 µm FWHM. These intensities volumetrically excite solid-density matter at timescales shorter than a phonon period, and predominantly couple to certain atomic orbitals when the photon energy is chosen with respect to resonances or absorption edges (Vinko *et al.*, 2012 ▸; Sperling *et al.*, 2015 ▸; Yoneda *et al.*, 2015 ▸).

Using the nanofocus setup, the diffraction-limited spot sizes and resulting intensities for a stack of Be CRLs with radii of curvature of 50 µm and 300 µm geometric aperture, a monochromatic (seeded) beam at 9 keV, 500 µJ pulse energy (accounting for the upstream beamline transmission) are as given in Table 3 ▸.

While the achievable intensities are ten times higher than using CRL3, the excited volume is small and comparable with the mean free path of photo- and Auger electrons which will rapidly redistribute the deposited energy. The photon-energy-dependent transmission can be calculated by equation (48) of Lengeler *et al.* (1999 ▸) and depends on the absorption properties of the lens material and the related effective numerical aperture.

A unique possibility of European XFEL is X-ray excitation of a sample with a MHz X-ray burst. Free-standing or statically compressed samples (*e.g.* in a DAC) can be sequentially and volumetrically heated and simultaneously probed by X-ray techniques (*cf.* Section 7).

While the delay between two subsequent pulses from the accelerator is limited to 222 ns, in the near future the HED instrument will also provide two-pulse techniques with separations <1 ps, either by two-color SASE with an electron chicane or by using an X-ray split-and-delay line (Mitzner *et al.*, 2008 ▸; Wöstmann *et al.*, 2013 ▸; Roling *et al.*, 2017 ▸; Kärcher *et al.*, 2021 ▸).

### 5.2. Diamond anvil cells   

The provision of X-ray photon energies >10 keV enables the HED instrument to perform experiments in DACs. The DAC setup in the IC1 chamber is suited for emission spectroscopy-type experiments (Section 6.2.6), whereas the one in IC2 provides a dedicated platform for MHz X-ray diffraction in dynamic DAC compression and heating experiments (Sections 6.4 and 6.4.1) which are described in detail by Liermann *et al.* (2021 ▸). High temperatures (typically in the range 1000–10000 K) are generated either via double-sided pulsed laser heating or by X-ray heating, the latter by varying the repetition rate of the X-ray pulses within a train. Dynamic compression is achieved in a piezo-driven dynamic DAC (dDAC) capable of compressing samples to megabar pressures on a millisecond time scale (Jenei *et al.*, 2019 ▸), enabling the study of material behavior under intermediate strain rates between those achieved in static and shock compression experiments. In dynamic, laser-driven shock compression experiments, DACs can also be used to precompress materials in order to reach higher pressures at moderate temperatures which are not accessible by shock-compressing materials from ambient pressures (Brygoo *et al.*, 2015 ▸; Loubeyre *et al.*, 2012 ▸).

### 5.3. Optical lasers   

#### 5.3.1. ReLaX laser   

The High-intensity Relativistic Laser at XFEL (ReLaX) is based on a commercial titanium sapphire laser system manufactured by Amplitude Technologies in France. The laser architecture follows a double chirped pulse amplification scheme, with temporal contrast enhancement using polarization filtering and spectral broadening by cross-polarized wave generation (XPW). It can deliver up to 300 TW at 5 Hz repetition rate and 100 TW at 10 Hz in nominal operation mode with pulses as short as 25 fs FWHM. The wavelength is (800 ± 40) nm. The whole laser chain has been designed with high redundancy of pump lasers and low fluence due to oversized optics in the compressed beam transport. The optical compressor, beam transport, and diagnostic package have been designed and manufactured through HIBEF by HZDR. Two loops on the wavefront using a deformable mirror are employed, one before and one behind the optical compressor. The commissioning and integration phase into the HED instrument was successfully completed in 2019. In addition to the main beam of ReLaX, low-energy probe beams are available, either for optical probing or timing cross-correlation with X-rays. The unfocused main beam diameter is 15 cm. Experiments with the ReLaX laser are only possible in IC1 (see Section 6.2), which is equipped with an *f*/*#* = 2 off-axis parabola as final focusing optics, allowing multiple sample irradiation geometries, *e.g.* co-linear, 45° or normal to the X-ray beam propagation direction. Sending the uncompressed pulse to IC1 was not foreseen and is currently technically impossible.

#### 5.3.2. DiPOLE 100-X laser   

The High Energy laser DiPOLE 100-X (Diode Pumped Optical Laser for Experiments) is an all diode-pumped 100 J class ytterbium:YAG based laser, manufactured by STFC CLF (Central Laser Facility) in the UK and the University of Oxford as part of the UK’s contribution to the HIBEF UC (Phillips *et al.*, 2019 ▸). The laser system delivers 100 J for a 10 ns pulse duration and 37 J for a 2 ns pulse duration at the fundamental wavelength (1030 nm). In addition it is capable of 10 Hz operation resulting in kW output of optical light. Since the main scientific use of DiPOLE 100-X is laser-driven shock and ramp compression, the laser provides pulse shaping capabilities with a resolution of 125 ps, allowing arbitrary waveforms ranging in duration from 2 to 15 ns. Frequency doubling of the 1030 nm laser to 515 nm with 60 J at 10 Hz for a 10 ns pulse has been demonstrated using a 60 mm large aperture lithium-triborate (LBO) crystal (Phillips *et al.*, 2021 ▸); conversion efficiencies for a 2 ns pulse are expected to be similar. The laser is synchronized with the X-ray beam using the XFEL timing system which has an RMS jitter of approximately 10 ps. The temporal total temporal jitter of the laser beam against the X-ray beam including all components is not yet characterized, but expected to be better than 50 ps. We also plan an online monitoring of the arrival time of both laser and X-rays. Phase plates providing flat-top focal spot profiles ranging from 100 to 500 µm diameter shall be provided. The DIPOLE 100-X laser will be available in both IC1 and IC2 with irradiation ranging from close to co-propagation (22.5°) to perpendicular.

**Figure 11:**
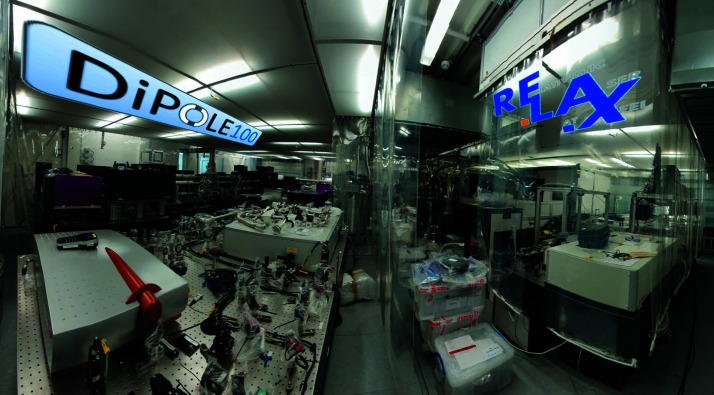
View into the HED laser bay with both HIBEF high-power lasers DIPOLE 100-X (left) and ReLaX (right).

The full capabilities of DiPOLE 100-X can be exploited in combination with the HIBEF-provided VISAR (see Section 6.7.1) system and the high-precision XRD platform at IC2 (see Section 6.4). The DiPOLE 100-X laser was delivered to European XFEL in 2019 and is currently being commissioned. The first user experiment is scheduled for 2022. Fig. 11 ▸ shows both the ReLaX and DiPOLE 100-X lasers in the laser bay.

#### 5.3.3. Pump–probe laser   

In addition to the HIBEF optical lasers, the pump–probe (PP) laser, developed at European XFEL (Palmer *et al.*, 2019 ▸), offers intense laser pulses tailored to the unique time structure of the XFEL bunch pattern. This laser, which operates with the non-collinear optical parametric amplifier (NOPA) scheme, has a central wavelength of 800 nm with a close to Fourier-limited bandwidth for pulses down to 15 fs duration. The laser can deliver synchronized pulses in 10 Hz bursts of up to 600 µs length and an intra-burst repetition rate up to 4.5 MHz. A maximum pulse energy of ∼2 mJ is available at a reduced intra-burst repetition rate of 100 kHz (Table 4 ▸). The pulse duration can be adjusted between 15 and 300 fs FWHM via bandwidth management while remaining close to the Fourier limit (no chirp). The PP laser is operational since 2020. Currently, the laser is operated with a single pulse energy of around 1 mJ with 100 kHz repetition rate and pulse duration of 17 fs FWHM. The second harmonic generation (SHG, λ = 400 nm) is also available. The conversion efficiency to SHG is only about 15% at 15 fs operation due to its large spectral bandwidth. In addition, the pump laser for the NOPA can be delivered to experiments upon request. This laser has a fundamental wavelength of 1030 nm with ∼2 nm bandwidth which results in ∼1 ps pulse duration, or ∼500 ps in chirped mode. This operation mode is particularly interesting for applications which require higher single pulse energies of up to 35 mJ.

#### 5.3.4. Synchronization of optical lasers and XFEL pulses   

**Figure 12:**
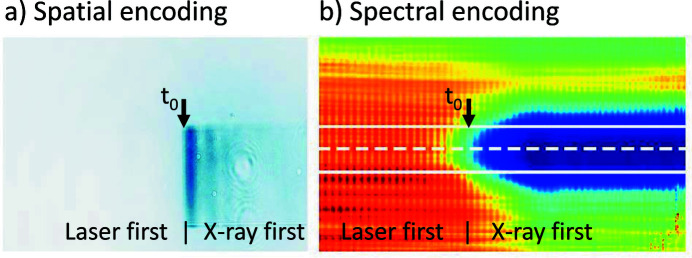
Typical images of PAM. (*a*) Spatial encoding: vertical versus horizontal position; time mapping results form an angle between the laser and the X-ray on the sample (2 µm-thick Si_3_N_4_). The temporal window (horizontal) is about 3.6 ps. (*b*) Spectral encoding image: lateral position versus the wavelength mapped to time by a chirped laser pulse collinear with the X-ray perpendicular to the sample (100 µm-thick YAG:Ce). The temporal window (horizontal axis of the image) is about 1.7 ps. Both show a shadow due to the X-ray-induced opacity change of the sample. At *t*_0_ both beams are timed. The horizontal edges in (*a*) result from the upstream partially closed power slits which are not influencing (*b*).

The optical lasers ReLaX, DiPOLE, and PP require spatial and temporal synchronization with the X-ray pulses which places high demands on environmental stability and an extensive online diagnostics. The linear accelerator provides pulses according to its own timing, and the various laser sources need to be synchronized relative to it. When lasers are synchronized using the accelerator’s radiofrequency only, a jitter of about 300 fs is observed (Kirkwood *et al.*, 2019 ▸). To improve on this, a Master-Laser-Oscillator (MLO) provides a more stable timing reference. A dispersion-compensated, actively stabilized optical fiber link synchronizes the individual oscillators of the ReLaX and PP laser on a sub-10 fs level. Nevertheless, the subsequent amplification process in both the FEL and the optical lasers and their long beam transport will lead to additional timing jitter and long-term temporal drift. Therefore, a pulse-resolved online photon arrival monitor (PAM) is implemented to record the relative arrival time between the optical and the X-ray pulses at the 10 Hz repetition rate. The PAM is permanently installed about 10 m upstream of the IC1 sample position in the optics hutch, before the X-ray attenuator and CRL3. This allows the jitter measurements to be quasi-independent from the X-ray focusing scheme and attenuation level which may be varied during experiments. The obtained data can be later used for time-sorting and binning. The typical sample is Si_3_N_4_ with 2 µm thickness. This ensures a high X-ray transmittance (>90% at >5 keV and >97% at >8 keV) for experiments downstream. The PAM consists of two sample chambers which allows simultaneous measurements using spatial (Harmand *et al.*, 2013 ▸; Riedel *et al.*, 2013 ▸) and spectral encoding (Bionta *et al.*, 2011 ▸) (Fig. 12 ▸) methods. While spatial encoding provides a better signal-to-noise ratio, the spectral encoding appears to be more robust against X-ray pointing fluctuations. To ensure that PAM works at different X-ray photon energies, Si_3_N_4_ of 4 and 6 µm thickness and a high-*Z* material sample (*e.g.* YAG:Ce 10, 20 µm thicknesses) are available, and are interchangeable during experiments.

The arrival timing jitter between the X-ray and the PP laser on PAM was measured to be 20–30 fs RMS.

### 5.4. Pulsed magnet   

In 2021, the HIBEF UC will install a 750 kJ capacitor bank with a peak current of 100 kA and pulsed magnets in different geometries: a horizontal bi-conical 60°–20° solenoid with peak fields of 60 T, and a split-coil (30 T) for diffraction experiments exploiting the full equatorial plane. Both coil systems integrate an eddy current shield in order to minimize stray fields and resulting vibrations due to interactions with the environment. The coils are cooled by liquid-nitrogen bath cryostats and the sample cryostat provides temperatures between 4 K and 600 K. The current design of the pulsed magnet setup represents a separate experimental platform optimized for peak field strengths and cryogenic temperatures. Thus, it cannot be used in conjunction with other drivers such as DACs or optical lasers. Furthermore, development of a phase retarder to control the polarization of the incident X-ray beam and of the polarization analyser for the scattered X-rays is currently ongoing.

## 6. Experimental platforms   

### 6.1. Experimental hutch layout   

The experiment hutch enclosure is constructed from up to 100 cm-thick heavy concrete walls, to anticipate the radiation and particles generated from the intense optical laser–matter interaction (*e.g.* using the ReLaX laser) and has been designed based on FLUKA simulations (Nakatsutsumi & Tschentscher, 2013 ▸; Battistoni *et al.*, 2015 ▸). Transport of large equipment in and out of the hutch is possible through a 3 m-wide by 2.5 m-high sliding door.

**Figure 13:**
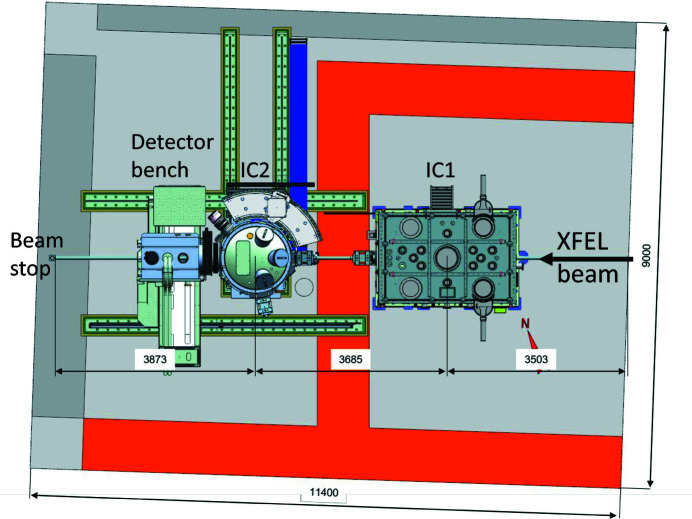
Floor plan of HED experiment hutch with the positions of IC1 (right, rectangular), IC2 (center, round) and the AGIPD on the detector bench on the left. The XFEL beam enters from the right. The dark-gray areas indicate hutch infrastructure, while the orange areas mark access paths. Dimensions are given in millimetres.

The floor plan of the 4 m-high HED experiment hutch is shown in Fig. 13 ▸, and the X-ray beam path is 1400 mm above the hutch floor. The hutch is organized in two interaction areas (IAs): the X-rays enter IA1 by passing through a differential pumping section into the fixed interaction chamber (IC) 1 (see Section 6.2) and then proceed to IA2. All drivers except the pulsed magnet can be brought to the IC1 chamber. Furthermore, the ReLaX (Section 5.3.1) and DiPOLE (Section 5.3.2) laser beams are brought into the experiment hutch from the laboratory on the top floor through chicanes in the roof. On either side of the IC1 chamber are optical tables for optical laser diagnostics and optical sample diagnostics such as VISAR (Section 6.7.1).

The IA2 allows for various dedicated setups. Here, the IC2 chamber (Section 6.4) is permanently mounted on a rail system, which is embedded in the hutch floor, and can be brought into the beam, or parked by the wall. In the near future, IA2 will also host a goniometer for pulsed magnetic field experiments.

Downstream of IC1, rails run parallel to the X-ray beam path, carrying a 3 m-wide detector bench. It can be located at any position between the rear end of IC1 and the beam stop at the hutch wall. The bench is optimized to minimize vibrations and equipped with spectrometers and detectors including VAREX or AGIPD (see Sections 6.4.4 and 6.4.3).

The standards for vacuum in the experiment chambers require clean conditions at <10^−4^ mbar pressure (Schmidt & Dommach, 2015 ▸). This volume is decoupled from the ultra-high vacuum in the optics hutch and tunnels (∼10^−9^ mbar) by a differential pumping stage (DPS) which allows window-less X-ray operation. However, if the experiment requires ambient pressure, gate valves before IC1 and IC2 can be used to separate the chambers which are equipped with a 10 mm-diameter, 100 µm-thick diamond window with excellent X-ray transmission.

### 6.2. Interaction chamber 1   

IC1 is a large multi-purpose chamber, manufactured by TOYAMA. All laser drivers, a manifold of sample geometries, spectrometers and other diagnostics can be arranged in vacuum around the X-ray beam at the TCC. The chamber body is made from an Al alloy to avoid long-duration nuclear activation during laser-plasma experiments which could interrupt user operation. Perpendicular to the X-ray path, large hinged doors allow easy access. The primary access doors open into a clean tent providing a flow of filtered air.

**Figure 14:**
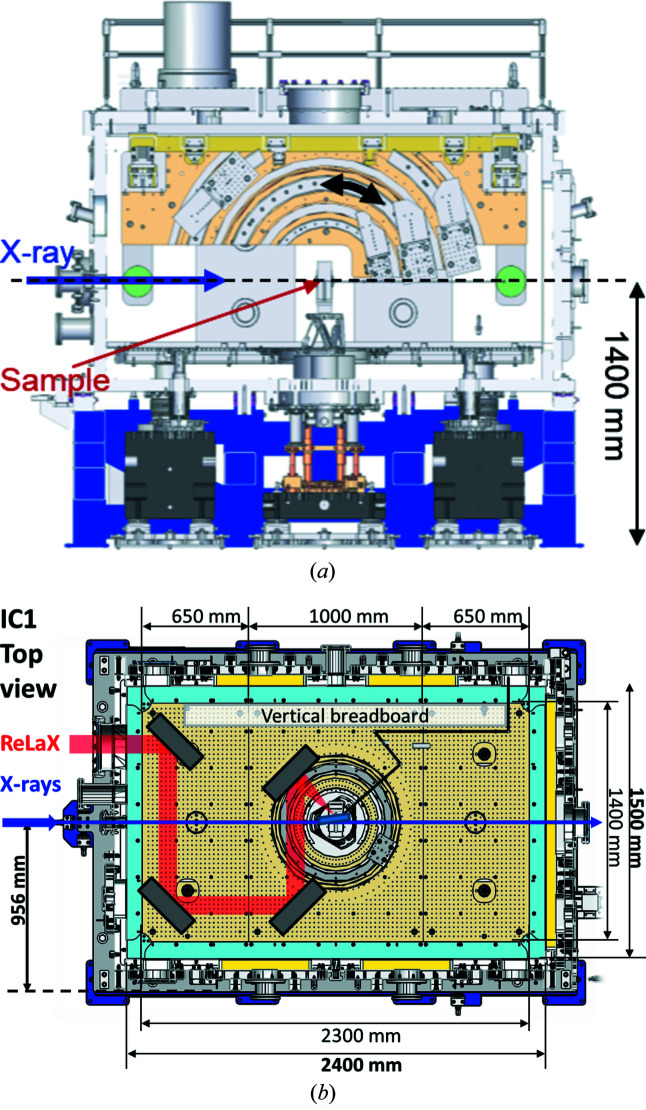
(*a*) Cut through the schematic of the IC1 target chamber showing the vertical breadboard with circular rails. (*b*) Top view of the interior of IC1; the access doors are at the bottom. In red, a typical beam path for the ReLaX laser is shown, and the back line indicates a sample viewing system. The beam transport of the DiPOLE-100X high-energy laser in IC1 is flexible and customized configurations are currently evaluated.

As the X-rays at European XFEL are horizontally polarized, scattering is preferably measured in the vertical plane where the scattered signal is not reduced by polarization effects. Therefore IC1 provides a vertical breadboard with motorized rails in the shape of circular arcs with the TCC in their centers (see Fig. 14 ▸). The arc radii are *R* = 306, 517 and 750 mm. Onto these, motorized carriages are mounted which can hold detectors and spectrometers of several kg weight. A central target mount and a horizontal breadboard are mechanically decoupled from each other and the vacuum chamber itself, and rest on separate granite supports. The dimensions of the horizontal breadboard are 2.3 m × 1.4 m, and the X-ray beam is (349 ± 1) mm above its surface. It provides M6 threaded holes in a 25 mm pattern. The IC1 walls are equipped with multiple feedthroughs with Japanese Industry Standards (JIS), ISO-K, and KF standards. On the roof of IC1, six turbomolecular pumps (HIPACE 800, Pfeiffer) with a pumping power of 790 l s^−1^ (for N_2_) pump the chamber through two large gate valves to <10^−5^ mbar.

#### 6.2.1. Sample tower   

**Figure 15:**
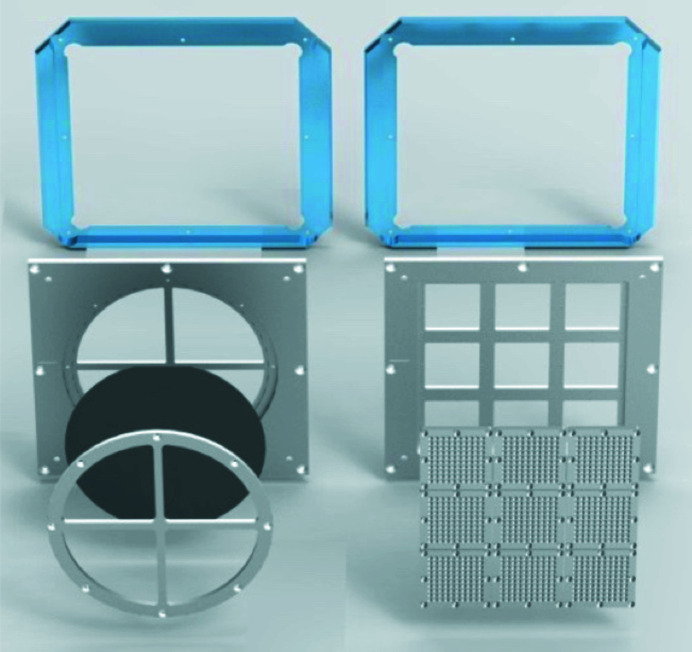
EUCALL standard frame design available at HED. The outer (blue) frame is unique to HED. The inner-frames (in gray/silver) can be customized for each experiment. This standard is employed at other beamlines at the EuXFEL, as well as other facilities such as ESRF and ELI beamlines.

In the center of IC1, an electrically and mechanically insulated sample tower consists of (from top to bottom): horizontal and vertical linear stages (Fast Sample Scanner), Hexapod (H-824, Physik Instrumente), 360° rotation stage (Goniometer 411, Huber Diffraktionstechnik), and a linear Y-axis (height) stage. It is possible to remove the Fast Sample Scanner to directly access the Hexapod. The scanner accommodates EUCALL (Appleby *et al.*, 2017 ▸; see also Prencipe *et al.*, 2017 ▸) standard sample holders and its travel ranges allows for 10 cm × 10 cm effective sample area available (see Fig. 15 ▸). Within these limits, the sample mount can be freely customized to the shape, thickness, structure and orientation as required. A description and specification for each stage is summarized in Table 5 ▸.

#### 6.2.2. Sample exchanger   

Frequent sample replacement is required due to the high repetition rate of both the X-ray beam and the optical laser systems. To mitigate the time for venting and pumping processes (20–30 minutes each), a robotic sample exchange system was constructed, consisting of a motorized sample exchange arm and a load-lock chamber on top of IC1, which is only compatible with EUCALL standard frames.

This system allows for the replacement of a frame mounted on the fast sample scanner, with one from the cassette in the load-lock, without breaking the vacuum. Each cassette can hold up to 16 sample frames with an overall thickness (including mounts/frames) <10 mm. For thicker samples, fewer frames may be stored accordingly. This system is currently under commissioning.

#### 6.2.3. Microscopes   

*Questar.* Two long-distance microscopes (QM 1 MKIII, Questar Cooperation) are mounted externally to IC1, upstream and downstream from the sample. The focus is motorized. Both telescopes are positioned at a working distance of ∼1.5 m from the sample and have a field of view of ∼13 mm × 8 mm. They have a spatial resolution of 20 µm, with a sampling of about 6.5 µm per pixel. Motorized shutters at each microscope protect the optics and camera sensors from high intensity radiation generated during optical laser experiments.

*In-Line Microscope (ILM).* Two ILMs are mounted in IC1 on multi-axis translation stages. The upstream ILM consists of a 10× magnification microscope objective with 2 mm field of view, 33 mm working distance, and a 2.5 mm central hole which allows the incoming X-ray beam to pass through. The downstream ILM can accommodate standard objectives with various magnifications (*e.g.* 4×, 10× or 20×), to image the sample or characterize laser focal spots. The multi-axis stages can be moved on a horizontal circular rail around the sample, and can be adjusted to micrometere precision.

#### 6.2.4. Cryogenic liquid jet targets   

Liquid jets and cryogenic liquid jets have great potential to deliver replenishing targets for high-repetition-rate experiments without causing degeneration of laser optics by target debris (Kim *et al.*, 2016 ▸). Moreover, they provide solid density matter which under ambient conditions only exists in the gas phase, and hence give experimental access to scientifically interesting samples such as hydrogen, methane, water and helium (Obst *et al.*, 2017 ▸; Göde *et al.*, 2017 ▸). The cryogenic jet platform implemented in IC1 will issue well characterized jets of cylindrical and sheet geometries with micrometre position accuracy and <10 µm diameters. Depending on the pumping speed and the gas properties, the maximum flow is limited to rates of about 500 sccm (1 sccm = 0.016 mbar l s^−1^) which allow target cross-sections of up to 100 µm × 100 µm with 100 m s^−1^ flow velocities. Larger jets with lateral dimensions exceeding 10 µm will be realized using a jet dump, currently under development.

#### 6.2.5. X-ray spectrometers   

**Figure 16:**
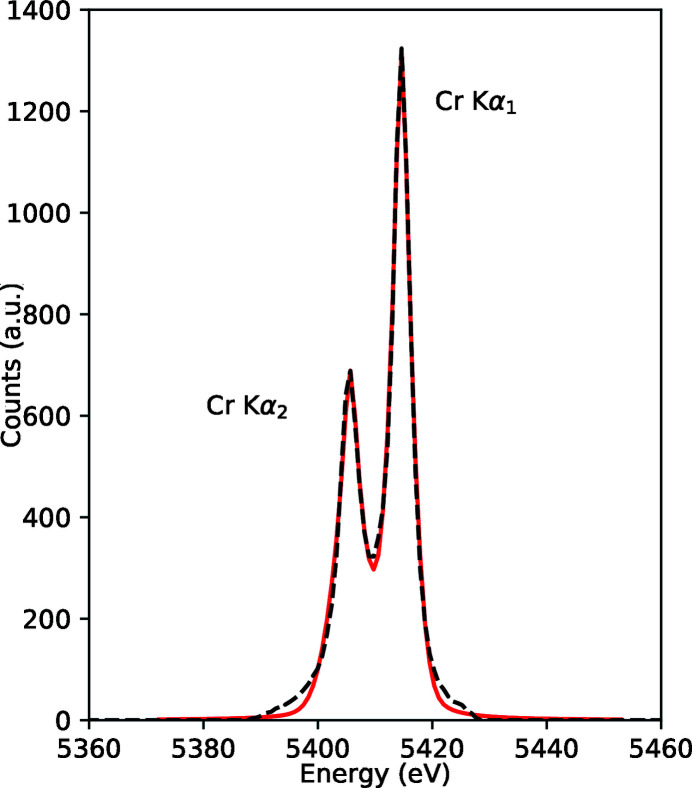
*K*α fluorescence of a 5 µm Cr foil irradiated by an 10 µm X-ray FEL spot for calibration purposes (black-dashed) with fit (red). The spectral broadening of the lines by the 80 mm radius-of-curvature and 40 µm-thick HAPG crystal is ∼2 eV. Reproduced from Preston *et al.* (2020 ▸).

**Figure 17:**
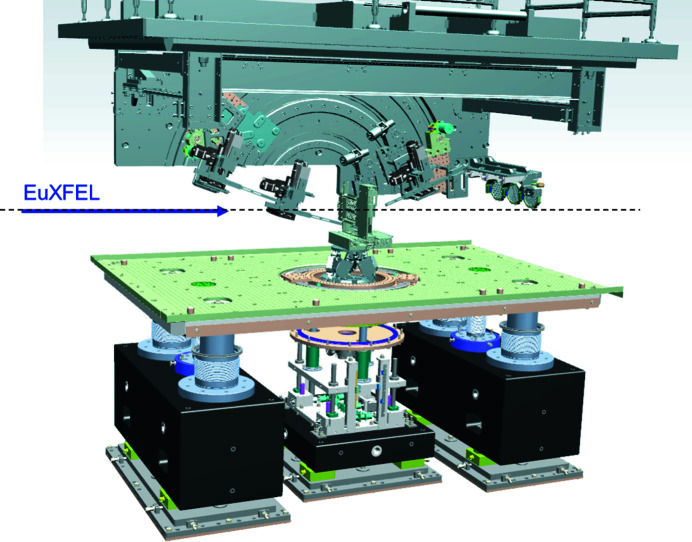
3D schematics of up- and downstream HAPG spectrometers and three diced analyzers mounted on the curved rail system in IC1, leaving the horizontal breadboard free for laser optics and further instrumentation.

Spectroscopy applications studying extreme states require vacuum conditions and sufficient flexibility for excitation methods and setup of the various technique-dependent spectrometers. In IC1, X-ray Emission Spectroscopy (XES) and Inelastic X-ray Scattering (IXS), both for meV and eV resolution, are provided. For in-vacuum use in IC1, we have designed highly efficient spectrometers (Preston *et al.*, 2020 ▸) using cylindrical mosaic crystals in von Hámos geometry (von Hámos, 1934 ▸). Highly Annealed Pyrolytic Graphite (HAPG) crystals (Zastrau *et al.*, 2013 ▸) with thicknesses of 40 µm or 100 µm or Highly Oriented Pyrolitic Graphite (HOPG) (Zastrau *et al.*, 2012 ▸) with thickness 100 µm can be used, with radii of 50 mm or 80 mm. The spectrometer is designed to use either an ePix100 or Jungfrau detector. The HAPG crystals have demonstrated (Preston *et al.*, 2020 ▸) to reach resolving powers of *E*/Δ*E* ≤ 2800 at photon energies between 5 and 10 keV. An example spectrum of Cr *K*α is shown in Fig. 16 ▸. Fig. 17 ▸ shows these spectrometers mounted in IC1, together with three diced analyzers (see below).

**Figure 18:**
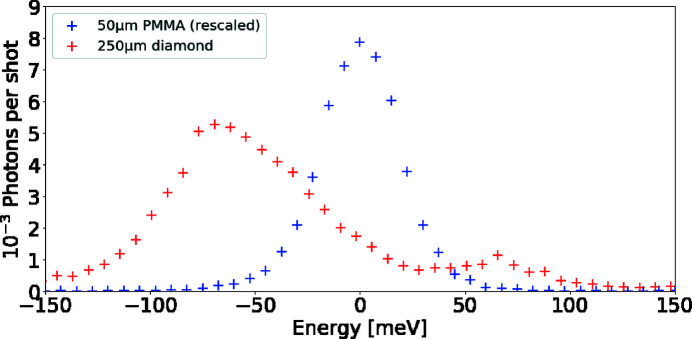
The spectra of the Si(533)-monochromated beam scattered from PMMA (elastic, blue) and single crystal diamond (inelastic, red) resolved with a diced Si (533) analyzer crystal. For better comparison, the elastic signal is reduced by a factor of ten. For diamond, the peaks correspond to phonon creation and annihilation. The spectrum is reproduced from Wollenweber *et al.* (2021 ▸).

Furthermore, in conjunction with the aforementioned high-resolution Si(533) monochromator (see Section 4.3), several diced analyzer crystals can be coupled with a detector to provide 40 meV spectral resolution at 7495 eV. As shown in Fig. 18 ▸, this is sufficient to resolve inelastic X-ray scattering from phonons in solids, or ion-acoustic waves in plasmas (Wollenweber *et al.*, 2021 ▸; Descamps *et al.*, 2020 ▸).

#### 6.2.6. Diamond anvil cell platform in IC1   

The DAC setup in IC1 is built for MHz spectroscopy at high pressure and temperatures. While static high pressures are generated by the DAC itself, high temperatures in IC1 are achieved via X-ray heating, by varying the repetition rate or intensity of the X-ray pulses within a MHz pulse train. Future developments of this experimental platform may include temperature measurements by optical diagnostics such as the portable system with streaked optical pyrometry (Section 6.7.2) and/or multi-color pyrometry (von Hámos, 1934 ▸). The X-ray spectrometer has been designed, built and contributed by the University of Dortmund, and is tailored for emission from matter contained in a diamond anvil cell. It comprises four vertically stacked analyzer crystals of either Si(111) or Si(531) with a radius of curvature of 250 mm and a size of 110 mm × 20 mm, covering an energy range of 6–8 keV with a resolution of 0.3–0.4 eV (Klementiev & Chernikov, 2020 ▸).

### 6.3. Small X-ray detectors   

The HED instrument is offering compact and vacuum compatible X-ray detectors. All detectors are suitable for X-ray diffraction, and can be used in conjunction with the aforementioned spectrometers. They can be positioned inside IC1 using the vertical breadboard and motorized translation systems. In air, the detector bench in IA2 can be used to achieve larger sample–detector distances of up to 7 m for small-angle X-ray scatting (SAXS), radiography, or phase contrast imaging (PCI).

**Figure 19:**
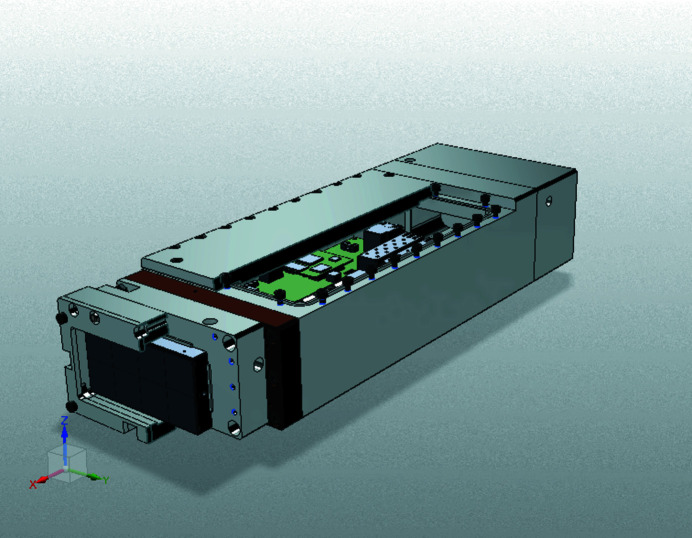
Model of the Jungfrau detector in an air-box, developed at the HED Instrument for in-vacuum operation.

The detectors inside IC1 are the ePix100 (Blaj *et al.*, 2016 ▸; Klačková *et al.*, 2019 ▸), ePix100H hammerhead (Blaj *et al.*, 2019 ▸), and the Jungfrau (Mozzanica *et al.*, 2018 ▸). Their specifications are summarized in Table 6 ▸. The HED instrument currently offers two vacuum-compatible ePix100 modules to users, while two more modules with hammerhead sensors are foreseen. A dedicated air-box housing developed at European XFEL (Fig. 19 ▸) enables also the Jungfrau detector to be operated in vacuum. The Jungfrau features automatic gain switching of each pixel which allows detection of single photon events and high signal levels of up to 10000 12 keV photons in a single image (Redford *et al.*, 2018 ▸, 2020 ▸). In total, four single Jungfrau modules are available and two modules can be combined into a larger detector with sensor size 2048 × 516 or 1024 × 1024 pixels with an inter-modular gap of 4.5 mm and 2.5 mm, respectively.

### 6.4. Interaction chamber 2   

**Figure 20:**
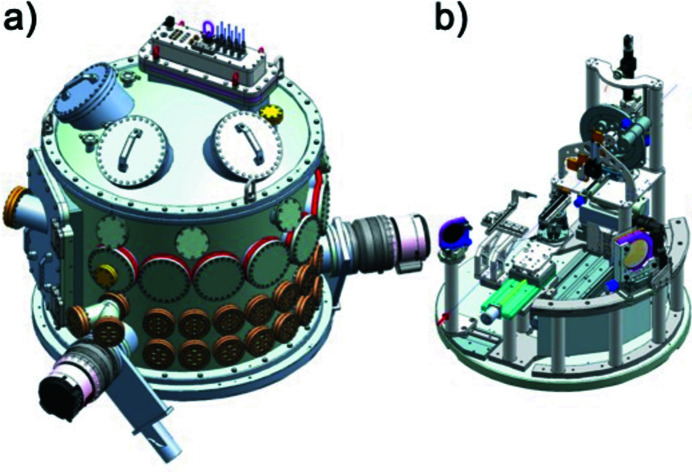
Interaction chamber IC2. (*a*) Vacuum chamber. (*b*) Platform for high-pressure research with diamond anvil cells.

**Figure 21:**
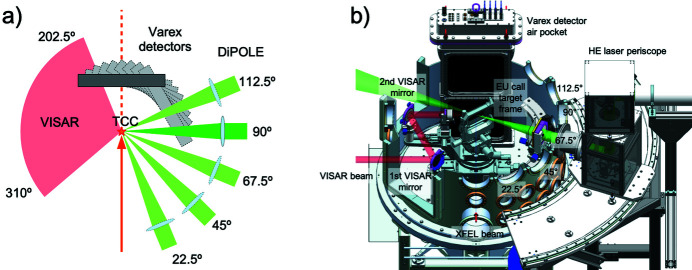
Dynamic laser compression platform optimized for flexible experimental geometries. For the drive laser, this is realized through five individual laser entrance ports at angles of 22.5°, 45°, 67.5°, 90°, and 112.5° with respect to the incident X-ray beam, to which the DiPOLE beam transport is coupled via an external periscope system. The VISAR beam enters the chamber through a single port and covers an angular range from 202.5° to 310° via a pair of fixed and mobile mirrors inside the chamber. The Varex detector system can be rotated around the interaction point in steps of 7.5°.

IC2 [Fig. 20 ▸(*a*)] is optimized for diffraction experiments, exploiting the high photon energies and MHz time structure of the X-rays for the study of materials under extreme pressures, temperatures and strain rates. It hosts two experimental platforms: one for DAC experiments [Fig. 20 ▸(*b*)], and one for dynamic laser compression experiments using the DiPOLE 100-X laser (Fig. 21 ▸).

The IC2 chamber has an outer diameter of 1360 mm and a height of 1520 mm. Further details are given by Liermann *et al.* (2021 ▸). The chamber is located in IA2 and can be moved between the operating and parking position on a rail system. The vacuum system allows for turnaround times below 30 minutes including venting, pumping and sample exchanges. IC2 can be coupled with either of two detector systems: the AGIPD 1M detector (Section 6.4.3), designed to resolve individual pulses at the minimum bunch spacing of the X-ray at 222 ns, and a twin configuration of two Varex flat-panel detectors (Section 6.4.4) for maximum gapless coverage at 10 Hz repetition rate in an EMP- and debris-resistant housing.

#### 6.4.1. Diamond anvil cell platform in IC2   

This DAC platform [Fig. 20 ▸(*b*)] is designed to explore MHz diffraction for X-ray heating, pulsed laser heating, and dynamic compression experiments using piezoelectric drivers. A sample stack provides all motorizations that are required to align a DAC in the center of rotation, relative to the detector and X-ray beam. Stability and resolution were optimized for studies of micrometre-sized samples with beam sizes down to 100 nm. Up to six conventional or three dynamic DACs can be mounted on carousel type sample exchangers. The DAC platform can be combined with a double-sided laser-heating setup which integrates sample observation, near infrared pulsed laser heating, and streaked optical pyrometry in a coaxial design. The addition of a four-channel multi-color spectrometer for fast, high-sensitivity pyrometry is planned.

#### 6.4.2. Dynamic laser compression platform   

The dynamic laser compression platform was conceived for maximum flexibility in the geometry between the DiPOLE laser, VISAR diagnostics (Section 6.7.1), and X-ray diffraction (Fig. 21 ▸). The sample stack was build around the EUCALL sample frame system. In addition, it features mounting interfaces for user-supplied sample delivery systems such as tape targets, or static precompression devices. For sample observation, Questar long-focal-distance microscopes aimed at the TCC can be installed on five ports at 0°, 34°, 79°, 124°, 280° in the horizontal, with an observation angle of 18° with respect to the equatorial plane.

#### 6.4.3. AGIPD detector   

**Figure 22:**
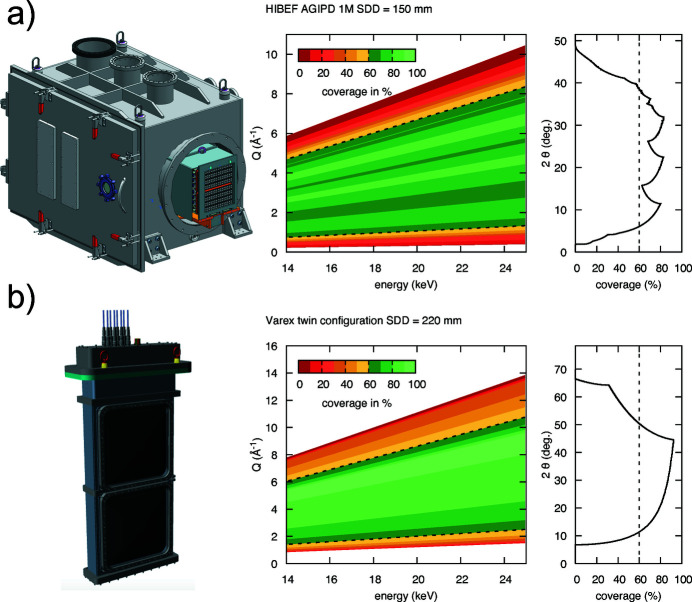
(*a*) HIBEF AGIPD 1M detector and coverage as a function of X-ray energy. (*b*) Varex twin detector system and coverage as a function of X-ray energy for the downstream position.

A dedicated 1 megapixel version of the Adaptive Gain Integrating Pixel Detector (AGIPD) (Allahgholi *et al.*, 2019 ▸) will be integrated in order to perform diffraction experiments at the X-ray pulse repetition rate of 4.5 MHz within a pulse train [Fig. 22 ▸(*a*)]. The AGIPD features automatic gain switching covering a dynamic range from single photon sensitivity (at 12 keV) up to 10^4^ photons per pixel. Up to 352 frames per train (with adjustable bunch pattern) are acquired in analog pixel-based storage and read out at the train repetition rate of 10 Hz. While the initial version will be delivered with a 500 µm Si sensor, DESY FS-DS is currently developing an electron collecting high-*Z* version for increased efficiency at high X-ray energies. The 1 megapixel version consists of sixteen 128 × 512-pixel modules in an 8 × 2 arrangement. The system is integrated in an independent vacuum chamber, which is connected to IC2 through a DN 500 gate valve and bellow system, in order to keep the detector cooled and biased when the interaction chamber has to be vented for sample exchanges. Inside its chamber, the detector is mounted on a positioning unit, in order to extend the sensor area into the interaction chamber for maximum coverage of up to 2θ = 40° at sample–detector distance (SDD) = 150 mm corresponding to *Q* = 9 Å^−1^.

#### 6.4.4. Varex detector   

The Varex twin-detector system is designed to provide maximum gapless coverage and high quantum efficiency at high X-ray energies for DAC and shock experiments in IC2 at the bunch-train repetition rate of 10 Hz [Fig. 22 ▸(*b*)]. These specifications are matched by two Varex XRD 4343 CT flat-panel detectors with scintillator panels consisting of CsI:Tl oriented needle crystals which are bonded to a 2880 × 2880 pixel (140 µm × 140 µm) TFT-diode array with an active surface of 432 mm × 432 mm. In order to reach 2θ angles of 64.5° in the vertical, the detectors need to be placed inside IC2 at a sample–detector distance of 220 mm. This is achieved by insertion of two of these detectors into an air pocket equipped with thin (400 µm) Al windows through a dedicated lid for IC2. A twin configuration with a horizontal gap in the equatorial plane was chosen in order to avoid parasitic scattering from a beamstop and provide direct access to the transmitted beam for additional downstream diagnostics (*e.g.* intensity monitors, SAXS, and PCI).

### 6.5. Goniometer   

A five-circle goniometer optimized for diffraction experiments in horizontal geometry with a load capacity of 300 kg is foreseen. It carries the magnet/sample cryostat assembly and can be reproducibly placed at IA2 by the use of kinematic mounts, alternatively to the interaction chamber 2. On the detector arm three different detector systems are planned: (1) an AGIPD module taking advantage of the pulse structure of the EuXFEL up to 4.5 MHz, (2) small pixel area detector module collecting diffraction data with higher angular resolution, and (3) analyser crystal for polarization-dependent scattering experiments.

### 6.6. Equipment operation under harsh conditions   

Both the JUNGFRAU detectors in IC1 and the VAREX detector in IC2 are designed to operate together with the high-intensity (IC1) or high-energy laser (IC1 and IC2). The interaction of these lasers creates electromagnetic pulses (EMP) and secondary radiation. The detectors are operated in a vacuum-tight metal housing which acts as a Faraday cage. The JUNGFRAU detectors in IC1 show stabile operation during laser-solid interaction at intensities 10^20^ W cm^−2^. The more sensitive ePIX100 and AGIPD detectors are currently not compatible with high-intensity and high-energy laser experiments.

### 6.7. Optical diagnostics   

In order to characterize the macroscopic state of dynamically compressed or excited matter, several optical diagnostics developed for the investigation of laser–matter interactions have been implemented. This is important for two reasons. First, the microscopic behavior of matter measured with X-rays can be related to macroscopic properties. Second, these diagnostics can measure the macroscopic evolution of the material over a large temporal and spatial scale, thus relating the point in time measurement of the X-rays with, for example, the compression history of a sample.

#### 6.7.1. Velocity Interferometer System for Any Reflector – VISAR   

In order to characterize laser-shocked samples, the HIBEF UC has contributed a VISAR system (Barker & Hollenbach, 1965 ▸). VISAR enables the measurement of shock or interface velocities ranging from less than 100 m s^−1^ to above 50 km s^−1^ with a temporal resolution better than 10 ps. The VISAR system has in total three parallel arms to cover a wide range of detectable velocities which can range from 0.3 km s^−1^ fringe^−1^ to 40 km s^−1^ fringe^−1^. Two arms operate at 532 nm and one at 1064 nm, permitting the simultaneous measurement of reflectivities at two different wavelengths. The system is able to record temporal windows as long as 50 ns and can adapt the field of view from 250 µm to 2 mm whilst keeping the full *F*/2.5 numerical aperture of the optical system.

The VISAR is available in both IC1 and IC2; the beam path from IC2 to the VISAR system is routed through IC1. In IC1, similar optics as in IC2 will be used to pick up the reflection from the target surface and thus allow to use the same VISAR system for experiments in IC1.

#### 6.7.2. Streaked Optical Pyrometry – SOP   

Streaked Optical Pyrometry (SOP) is an instrument to deduce the temperature of a sample by analysing its emitted optical radiation. The temperature is either deduced from fitting the spectral intensity distribution to gray body radiation or the absolute radiation intensity at a given wavelength. The latter method has higher sensitivity and provides spatial information but requires a reflectance measurement, which can be provided by the VISAR system. Using a streak camera as a detector permits recording the temperature of the sample over time spans ranging from 1 ns to 100 ms with the possibility of sub-ns temporal resolution. The SOP system can be used in both interaction areas for various experiments such as pulsed laser heating, shock compression or isochoric heating.

The SOP system specifically designed for IC2 detects thermal or fluorescence radiation emitted from the interaction of the XFEL pulses with a DAC sample, or during pulsed laser heating. The system features a HAMAMATSU streak camera with S-20 photocathode coupled to an optical spectrometer. It is placed outside the IC2, along with optics for high magnification microscopes for DAC sample observation, illumination, filtering and alignment. The spectral range for the optical spectrometer covers 440–850 nm.

#### 6.7.3. Fourier Domain Interferometry – FDI   

Fourier-domain interferometry (FDI) (Geindre *et al.*, 1994 ▸) enables the measurement of polarization-resolved reflectivity, phase shifts in the reflected probe due to plasma expansion or due to changes in refractive index via electronic excitation. The HED FDI system is available to users and consists of a 1:1 imaging spectrometer with an upstream Mach-Zehnder interferometer. It allows to measure the velocity of the critical density surface with ∼10 nm and a few 100 fs resolution in a ∼10 ps temporal window.

## 7. Methods and experiments   

The HED instrument supports a variety of ultrafast X-ray methods as summarized in Appendix *A* [App appa]. XRD can be realized by positioning in-vacuum detectors in IC1 at the required geometry to cover dedicated scattering angular ranges. Designed for precision diffraction, IC2 provides larger detectors in forward-scattering, which in combination with hard X-rays allows radial patterns for particle distribution functions or texture analysis to be recorded.

In general, IC1 with its larger volume and two breadboards is optimized for spectroscopic applications. The HAPG spectrometers (Section 6.2.5) can be used for both XES and IXS. We also provide perfect crystals for a dedicated von Hámos analyzer for measuring *K*-shell emission from the 3*d* transition metals Cr, Mn, Fe, Co, Ni, Cu and Zn. The spectrometer can be combined with a DAC sample environment and isochoric X-ray heating, which opens up the unique possibility to probe the electronic state at high pressures and temperatures of solid and liquid materials, *e.g.* that are of geological relevance.

X-ray imaging is achieved by placing a nanometre X-ray focus upstream of the sample (by using the nanofocusing CRL4) and an on-axis projection onto a detector. The distance to the target needs to be sufficiently large for magnified PCI with high spatial resolution. The imaging performance depends also on several other parameters such as X-ray wavelength, focus and feature size, and sample properties (Schropp *et al.*, 2015 ▸; Hagemann *et al.*, 2021 ▸). Both IC1 and IC2 qualify for PCI; however, IC1 allows for more propagation between sample and detector.

In order to illustrate the capabilities of the HED instrument, we highlight a few showcase experiments.

### 7.1. X-ray heating in DACs   

Increasing the temperature in samples under pressure solely by exposing them to the MHz X-ray pulse train (Meza-Galvez *et al.*, 2020 ▸) is an interesting alternative to the resistive or laser-heated DAC experiments (Spiekermann *et al.*, 2020 ▸) because of the possibility to suppress chemical reactions and contamination of the sample from the diamond anvils. High-precision diffraction experiments IC2 coupled with simultaneous SOP provided the temperature evolution and verified sample stability. The DAC assembly proved to be robust in the XFEL beam, which will in future enable novel pulse-resolved diffraction experiments at extreme conditions including high strain rates using MHz detectors.

**Figure 23:**
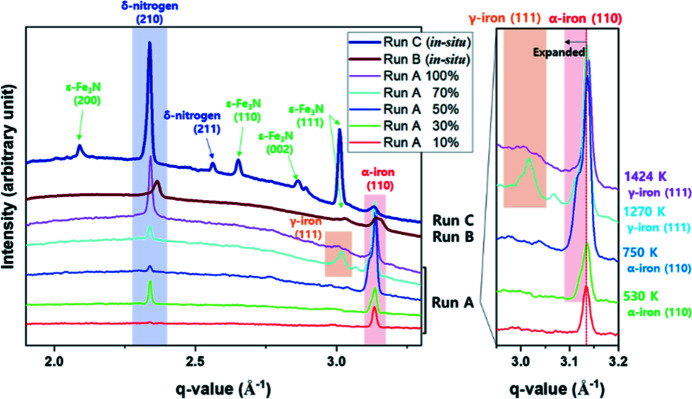
The changes of X-ray diffraction patterns of iron and nitrogen during *in situ* chemical reaction by XFEL pump-and-probe. An Fe foil surrounded by N_2_ was pre-compressed to 5 GPa in a DAC. The percentages in run *A* indicate the transmission (fluence) of the XFEL. At 100% XFEL transmission, run *B* contains 20 consecutive pulses while run *C* has consecutive pulses over 11 s. For more details and figure credit, refer to Hwang *et al.* (2021 ▸).

Recently, Hwang *et al.* (2021 ▸) observed the ultrafast synthesis of ɛ-Fe_3_N_1+*x*_ in a DAC from Fe and N_2_ under pressure using serial exposures of 17.8 keV pulses, using the IC2 DAC platform. When the sample at 5 GPa was irradiated by a pulse train separated by 443 ns (2.2 MHz), the estimated sample temperature at the delay time was above 1400 K, confirmed by *in situ* transformation of α- to γ-iron (Fig. 23 ▸).

### 7.2. Combined spectroscopy and diffraction experiments in DACs   

Differently to IC2, the DAC setup in IC1 was used in a number of measurements that combined emission spectroscopy [at the Fe-emission lines (Lin *et al.*, 2005 ▸)] and X-ray diffraction at 13 keV incident XFEL photon energy. The goal of the experiment was to study simultaneously the structure and electronic configuration of Fe-bearing compounds at extreme pressures and temperatures *in situ*. While under pressure, the sample was subjected to trains of several X-ray pulses which step-wise heated the sample in the DAC. During this temperature increase, the sample undergoes a spin cross-over (Cerantola *et al.*, 2015 ▸), which was detected both in diffraction and emission signals.

### 7.3. Laser shock compression experiments on planetary materials   

Geodynamic models of planetary interiors depend on the precise knowledge of the equation of state and the high-pressure phase diagram of materials such as SiO_2_ and MgO. Previous studies demonstrated in shock compression experiments that these materials can transition from electrically insulating solids to conducting liquids at ultra-high pressures (Umemoto *et al.*, 2006 ▸; Millot *et al.*, 2015 ▸) which suggest, for instance, that planetary structure models need to include multiple layers of conductive fluids.

The use of the DiPOLE 100-X laser system will allow investigating matter at extreme conditions by laser-induced shock- and ramp-compression. It will be possible to investigate the model systems SiO_2_ and MgO at pressures of up to several megabars at high or moderate temperatures with temporal pulse-shaping capabilities, which will consequently enable quasi-isentropic compression of material, reaching Hugoniot and off-Hugoniot high-pressure states.

The VISAR and SOP diagnostics will enable measurements of the pressure- and temperature evolution within the sample during shock transit, using a LiF or Al_2_O_3_ pressure window. Probing the materials during shock loading with the highly coherent and intense X-ray beam will permit the collection of time-resolved information about their respective density and crystal structures during compression.

### 7.4. Diagnosing relativistic laser plasmas   

Small-angle X-ray scattering (SAXS) allows the detection of nanoscale density fluctuations. Typically, the SAXS signal from laser-excited plasmas is expected to be dominated by the free-electron distribution (Kluge *et al.*, 2014 ▸); however, the fully tunable low energy-bandwidth XFEL pulses can be exploited for resonant SAXS (Kluge *et al.*, 2016 ▸). Here, ionic scattering becomes visible when the incoming X-ray photon is in resonance with a bound–bound electronic transition. In this case, the scattering cross-section dramatically increases so that the signal of X-ray scattering from ions is silhouetted against the free-electron scattering background which allows the measurement of opacity and derived quantities with high spatial and temporal resolution.

In the harsh environment of high-intensity laser inter­actions, intense secondary radiation and high-energy particles are generated, and the SAXS signal may suffer a significant increase of noise. To mitigate the noise, a mosaic graphite crystal that works as a Bragg mirror for the SAXS signal between 8 and 9 keV is developed, which allows detecting the signal behind an appropriate shielding (Šmíd *et al.*, 2020 ▸).

In addition, a solid target irradiated by a high-intensity laser pulse can become relativistically transparent, which then allows it to sustain an extremely strong laser-driven longitudinal electron current. The current generates a filament with a slowly varying Mega-Tesla level azimuthal magnetic field that has been shown to prompt efficient emission of multi-MeV photons in the form of a collimated beam required for multiple applications. At the HED instrument, these short-lived transient magnetic fields can be diagnosed via Faraday rotation (Wang *et al.*, 2019 ▸).

### 7.5. Grazing-incidence X-ray scattering (GIXS) to track surface and subsurface density dynamics at nanometre resolution   

The interaction between high-power, sub-ps lasers with metals or high-density plasmas occurs within the skin depth of the overcritical plasma, where the laser wave is evanescent. Therefore, the laser–plasma coupling relies on the details of the surface nano-structure within the skin layer, which amounts to a few tens of nm for solids. For a clear understanding of the complex physics involved in laser–matter coupling and subsequent energy transport, phase transition and surface expansion or ablation, it is crucial to visualize the surface and sub-surface density profile with nanometre and sub-ps resolution. X-rays become surface-sensitive in a grazing-incidence geometry, *i.e.* close to the critical angle for external total reflection which is typically below 1° for solids in the hard X-ray regime. In particular, grazing-incidence X-ray scattering (GIXS), which blocks the intense specular reflection and looks at the diffusely scattered 2D pattern, provides a wealth of information about the depth profile as well as the surface roughness and its correlation properties (Holy *et al.*, 1993 ▸; Müller-Buschbaum, 2003 ▸; Roth, 2016 ▸). The first grazing-incidence X-ray scattering of a femtosecond XFEL beam combined with a high-intensity femtosecond laser has been successfully demonstrated at the SACLA XFEL in Japan (Randolph *et al.*, 2020 ▸).

### 7.6. Probing the vacuum polarization   

Vacuum in the presence of strong electromagnetic field contains pairs of short-lived quantum particles that can act as an electric dipole, which may in turn change properties of the background field, a process called vacuum polarization (Karbstein & Mosman, 2019 ▸). To detect vacuum polarization effects, the probing light, in this case the XFEL beam, has to be linearly polarized with a high degree of purity (Schlenvoigt *et al.*, 2016 ▸). A specially designed highly optimized channel-cut polarizer and analyzer, each reflecting the beam six times inside the channel, has been shown to suppress residual light polarized in the orthogonal state to more than 11 orders of magnitude (Grabiger *et al.*, 2020 ▸; Bernhardt *et al.*, 2020 ▸). Such polarization purity is unprecedented for X-ray sources and opens up new opportunities for quantum optics and polarimetric experiments at XFELs.

### 7.7. Structure, electronic, and magnetic properties in pulsed high magnetic fields   

One of the open questions in underdoped cuprate high-*T*_c_ superconductors is the nature and connection of the different charge density wave states with the upper critical field. To study the emergence of superconductivity from these phases, diffraction from the weak charge density wave peaks in very high magnetic fields is required (Gerber *et al.*, 2015 ▸). Indeed, suppressing electronic order may provide a more general route to find new types of superconductors in various families of materials (Basov & Chubukov, 2011 ▸). The suppression of electronic order in high magnetic fields can also result in quantum criticality. Quantum critical matter in high magnetic fields may not only exhibit superconductivity but also electronic nematic phases (*e.g.* Fe-Pns) or so-called hidden orders, *e.g.* URu_2_Si_2_ (Mydosh & Oppeneer, 2011 ▸). In frustrated magnetic materials, diffraction allows the detection of broken structural symmetries accommodating a variety of magnetic phases, and magnetic scattering will make it possible to probe their order. Example systems where a magnetic field successively stabilizes a variety of novel exotic states (Ueda *et al.*, 2017 ▸) are the rare-earth iridates of the *A*_2_ *B*_2_O_7_ pyrochlore structure. Here, resonant magnetic X-ray diffraction with polarization control and analysis could be used to probe the interplay between the rare earth and Ir, in order to understand the quantum metal–insulator transition.

## 8. Conclusion   

We present the status of the HED instrument at the European XFEL after the first two years of operation. The performance of the baseline instrumentation, short-term future developments, and technical infrastructure are discussed. Experimental platforms and their diagnostics offered at the HED instrument are presented and first scientific results are highlighted.

## Figures and Tables

**Figure 24:**
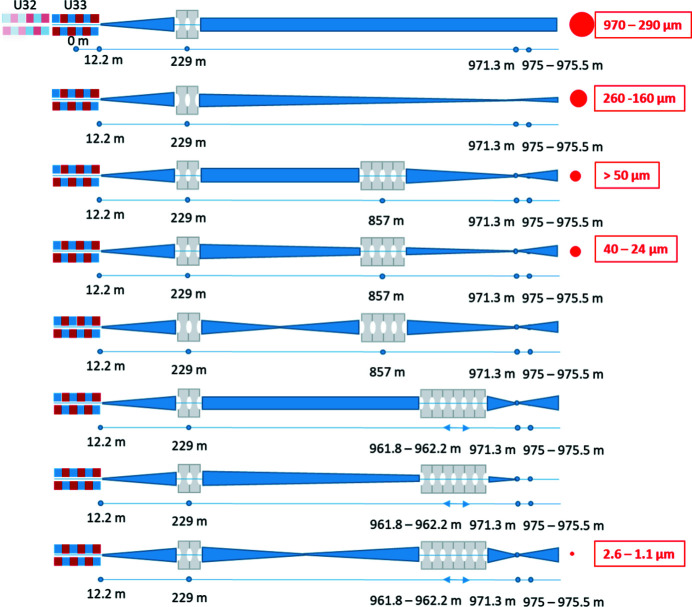
HED focusing optics. The X-rays enter from the left. The coordinate system starts at the center of U33. On the right, in red, are the achievable best focus conditions for 5–25 keV for monochromatic (seeded) beam.

**Table 1:** SASE2 undulator divergence (measured with imagers) and source size (calculated from divergence) for 0.25 nC bunch charge

Linac electron energy (GeV)	SASE photon energy (keV)	Source divergence (µrad)	Source size (µm)
11.5	6.0	2.44 ± 0.25	∼43
14.0	9.0	2.33 ± 0.25	∼30
16.5	17.8	1.68 ± 0.25	∼21

**Table 2:** Calculation of the Rayleigh length of the monochromatic beam and focal spread which includes a 0.3% FWHM spectral bandwidth using CRL1 intermediate focus and CRL3 tight focus Note that a diffraction-limited focus can only be achieved up to 20.5 keV at the TCC of IC1 – beyond that the refractive power of the installed lenses is not sufficient. Therefore, a pre-focusing with CRL1 combined with CRL3 focusing at the IC2 chamber is shown. A tight focus above 20.5 keV is possible by replacing some weaker lenses.

SASE photon energy (keV)	Diffraction limit focus (µm)	Rayleigh length of monochromatic beam (mm)	Rayleigh length with 0.3% bandwidth (mm)
6.0	1.0	4.1	28.5
8.0	0.8	3.0	28.5
9.0	0.8	3.8	29.5
13.0	0.3	0.9	27.5
18.0	0.3	1.0	28.2
25.0	0.7	8.3	43.6

**Table 3:** Transmission, diffraction limit and intensity for the nanofocus CRLs

	Transmission (%)	Diffraction limit (nm)	Intensity (10^18^ W cm^−2^)
20 CRLs	50	122	2.1
30 CRLs	37	89	3.0
40 CRLs	28	73	3.3
50 CRLs	22	64	3.4

**Table 4:** Basic specifications of the pump–probe laser

Name	Central wavelength (nm)	Repetition rate	Pulse duration (FWHM)
Mode 1 (NOPA)	800	100 kHz @ 2 mJ	15–300 fs nearly transform limited
		4.5 MHz @ 0.05 mJ	
Mode 2 (amplifier for NOPA)	1030	100 kHz @ 35 mJ,	0.9 ps (compressed), 500 ps (chirped)
		4.5 MHz @ 1 mJ	

**Table 5:** Specifications of the sample tower

Name	Travel range	Speed	Repeatability
Fast Sample Scanner horizontal	62.5 mm	0.1–20.0 mm s^−1^	2 µm
Fast Sample Scanner vertical	57.5 mm	0.1–15.0 mm s^−1^	2 µm
PI Hexapod horizontal	±22.5 mm	1 mm s^−1^	0.1–0.5 µm
PI Hexapod vertical	±12.5 mm	1 mm s^−1^	0.1–0.5 µm
PI Hexapod rotation (yaw, pitch)	±7.5°	11 mrad s^−1^	3 µrad
PI Hexapod rotation (roll)	±12.5°	11 mrad s^−1^	3 µrad
360° rotation stage	360°	N/A	<10 µrad
Height adjustment stage	150 mm	N/A	<0.3 µm

**Table 6:** Detector specifications available at IC1

Detector name	Pixel size (µm)	Noise (adu)	Gain (eV adu^−1^)	Dynamic range	Sensor size (pixels)
ePix100	50 × 50	<4	54	100 photons @ 8 keV	704 × 768
ePix100H	25 × 100	<4	54	100 photons @ 8 keV	176 × 1536
Jungfrau	75 × 75	<15	20	10^4^ photons @ 12 keV	512 × 1024

**Table 7:** Overview of X-ray methods supported at the HED scientific instrument

Method (acronym)	Accessible scientific parameters	Chamber platform
X-ray diffraction (XRD)	State (crystalline, amorphous, liquid)	IC1, IC2
Wide-angle X-ray scattering (WAXS)	Lattice spacing, density, crystallography, texture, strain, pair-distribution function (PDF)	IC2
Small-angle X-ray scattering (SAXS)	Nanometre structures	IC1, optional: HAPG analyzer (8 keV)
Grazing-incidence X-ray scattering (GIXS, GISAXS, GIWAXS)	Statistical properties (*e.g.* roughness and correlation lengths) of surfaces and interfaces, density and structural phase at the surface	IC1
X-ray emission spectroscopy (XES)	Element sensitivity, *K*- and *M*-lines	IC1, HAPG spectrometers
From plasmas	Ionization state of plasmas	DAC von-Hamos
From DACs	Electronic configuration, spin state	Spectrometer
Inelastic X-ray scattering (IXS)		IC1
Compton (back)-scattering	Ionization state of plasmas	HAPG spectrometers
Plasmon scattering	Electronic configuration	DAC von-Hamos
High-resolution IXS (meV)	Acoustic modes, ion temperature	Diced crystal analyzers
X-ray imaging		IC1, IC2
Phase-contrast imaging (PCI)	2D µm-resolved single-shot phase contrast image	Nanofocus CRLs
MHz imaging	Stroboscopic 222 ns 2D image sequence	MHz camera
X-ray polarimetry		IC1
Faraday rotation	Transient, strong magnetic fields	Bragg polarizers
Vacuum birefringence	Virtual e^−^–e^+^ pair plasma	

**Table 8:** A summary of the most important components with positions The coordinate system at EuXFEL is based on the center of the last undulator U33, which was by design assumed to be the source point.

XTD1/6	Distance (m)	XTD6	Distance (m)	OPT	Distance (m)	EXP	Distance (m)
Source point U33	0.0	ABS	306.6	OPT hutch wall	958	EXP hutch wall	967.8
Undulator end	12.2	COLA (65 mm × 25 mm)	307.6	PAM center	960	ALAS (center)	968.5
Transmissive imager	181.1	HIREX-II	380.8	Attenuators center	961	TCC IC1	971.3
SRA	188.4	PBLM	387.1	CRL3 center pos.	962.3	TCC IC2	975.0
KMONO	190.6	M3	390	IPM	965.8	IBS	980
COLB (17 mm diameter)	192.0	Imager (II-45)	400	OPT/EXP wall	967	End of EXP hutch	979.2
XGMin XTD1	199.6	Separation MID/HED	462.1				
ATT	227.6	PBLM	827.7				
CRL1	229	COLB (18 mm diameter)	828.5				
FEL Imager	242	SDL	841.0				
XTD1 end/XS2 start	245	BIU1	844.8				
XS2 end	277.2	MONO four-bounce	853.5				
XTD6 start	277.2	HR MONO	855.8				
M1	290	CRL2	857				
PBLM	298.0	BIU2	858.0				
M2	301.4	COLB (14 mm × 24 mm)	858.6				
MCP	302.9	XGM in XTD6	866.2				
Imager (II-45)	303.7	Pulse picker	877.7				
		COLA (30 mm diameter)	900				
		Imager (type I)	939.1				
		Front end	940.2				
		Wall end	942.6				

**Table 9:** Configurations of individual lenses (number and radii of curvature) in all installed CRL systems CRL 1, 2 and 3 consist of a so-called transfocator with ten rods which can be individually inserted. The term number refers to the amount of individual Be lenses in each rod. The apertures of these parabolic lenses relate to the maximum non-clipping illumination. The shown lens configuration in CRL3 can focus all photon energies from 5 keV up to 20.5 keV without any gap. If tight focusing of higher photon energies is required, either prefocusing with CRL1,2 needs to be used, or weaker lenses have to be replaced with stronger ones. However, the latter will result in gaps for focusing lower photon energies.

Rod	1	2	3	4	5	6	7	8	9	10
CRL1 lens radius (mm) × number	–	5.8 × 1	5.0 × 1	4.0 × 1	3.5 × 1	5.8 × 2	4.0 × 3	4.0 × 7	2.0 × 7	–
CRL1 apertures (mm)	4	3.8	3.53	3.16	2.96	3.8	3.16	3.16	2.76	4
CRL2 lens radius (mm) × number	5.8 × 1	5.0 × 1	4.0 × 1	3.5 × 1	5.8 × 2	5.8 × 4	5.8 × 7	4.0 × 10	3.5 × 10	2.0 × 8
CRL2 apertures (mm)	3.8	3.53	3.16	2.96	3.8	3.8	3.8	3.16	2.96	2.76
CRL3 lens radius (mm) × number	5.8 × 1	5.8 × 3	5.8 × 4	4.0 × 10	2.0 × 10	1.0 × 10	1.0 × 10	0.5 × 10	0.5 × 10	5.8 × 2
CRL3 apertures (mm)	3.8	3.8	3.8	3.16	2.76	1.95	1.95	1.38	1.38	3.8
CRL4 lens radius (mm) × number	0.05 × up to 50									
CRL4 apertures (mm)	0.3									

**Table 10:** Summary of each 2D imager, 10 Hz operation Invasive means that this imager is opaque to the X-ray beam, as opposed to an imager which is transmissive.

2D imager	Location from the source (m)	Type	Screens and thicknesses	Field of view (mm × mm)	Resolution (µm)
IMG-TR	190	Transmissive	YAG:Ce 25 µm, diamond 50 µm	25.6 × 19.2	35
IMGFEL	242	Invasive	YAG:Ce 100 µm, BN 60 µm, CVD 40 µm	17.62 × 23.5	15, 15, 59
POPInII45-1	303	Transmissive	YAG:Ce 100 µm	150 × 22.7	103
POPInII45-2	400	Transmissive	YAG:Ce 100 µm	148.4 × 22.1	112
SDLBM-1	845	Transmissive	YAG:Ce 1 mm	≫63 mm	≫200
SDLBM-2	858	Transmissive	YAG:Ce 1 mm	≫63 mm	≫200
HED-IMGPI	940	Transmissive	YAG:Ce 50 µm, CVD 40 µm	22.4 × 39.8	39.32
HED-BIU	960	Transmissive	YAG:Ce 25, 50, 100 µm	N/A	
HED-PAM	961	Transmissive	YAG:Ce 20, 100 µm	∼5 × 6	<10
HED-SBM	965	Transmissive	YAG:Ce 25 µm & 50 µm for beam split by BIU grating	N/A	
HED-Questar1	971.3	Transmissive	Variable	13 × 8	20
HED-Questar2	971.3	Transmissive	Variable	13 × 8	20
HED-IBS	978.5	Invasive	CVD 500 µm	N/A	

**Table 11:** Summary of each spectral analyser

Analyser name	Location	X-ray range	Crystal and bending radius	Resolution /spectral range (average)	Detector
HIREX-II	XTD6	5–25 keV	C(110), *R* = 100 mm	≤0.2 eV/300 eV	PhotonicScience
			Si(110), *R* = 50 and 100 mm		GOTTHARD I
			Si(111), *R* = 75 mm		
HED-flex	HED-EXP	5–20 keV	C(110), *R* = 100 m m	0.13 eV/100–200 eV	Zyla 4.2 Plus
			C(111), *R* = 75 mm		Zyla 5.5
			Si(110), *R* = 50–150 mm		GOTTHARD II
			Si(111), *R* = 50–150 mm		
CNRS-spec	HED-EXP	5–24 keV	C(110), *R* = 100 m m	≤0.3 eV/100–200 eV	Zyla 4.2 Plus
			C(111), *R* = 100 mm		Zyla 5.5
			Si(110), *R* = 50–150 mm		PI-MTE:2048B
			Si(111), *R* = 75 mm		GOTTHARD II

## Appendix A X-ray methods

An overview of the X-ray methods supported at the HED scientific instrument are given in Table 7 ▸.

## Appendix B Technical specifications

A summary of the most important components with positions is given in Table 8 ▸. The compound refractive lens schemes at HED is shown in Fig. 24 ▸, and the configurations of individual lenses are given in Table 9 ▸. Properties of X-ray imagers are given in Table 10 ▸, and details of the spectral analysers can be found in Table 11 ▸.

## References

[bb27] Abeghyan, S., Bagha-Shanjani, M., Chen, G., Englisch, U., Karabekyan, S., Li, Y., Preisskorn, F., Wolff-Fabris, F., Wuenschel, M., Yakopov, M. & Pflueger, J. (2019). *J. Synchrotron Rad.* **26**, 302–310. 10.1107/S1600577518017125 30855236

[bb88] Allahgholi, A., Becker, J., Delfs, A., Dinapoli, R., Göttlicher, P., Graafsma, H., Greiffenberg, D., Hirsemann, H., Jack, S., Klyuev, A., Krüger, H., Kuhn, M., Laurus, T., Marras, A., Mezza, D., Mozzanica, A., Poehlsen, J., Shefer Shalev, O., Sheviakov, I., Schmitt, B., Schwandt, J., Shi, X., Smoljanin, S., Trunk, U., Zhang, J. & Zimmer, M. (2019). *Nucl. Instrum. Methods Phys. Res. A*, **942**, 162324.

[bb6] Appel, K., Nakatsutsumi, M., Pelka, A., Priebe, G., Thorpe, I. & Tschentscher, T. (2014). *Plasma Phys. Control. Fusion*, **57**, 014003.

[bb73] Appleby, G. A. S., Pascarelli, D., Pahl, T., Tschentscher (2017). *Synchrotron Radiat. News*, **30**(5), 6–8.

[bb89] Barker, L. M. & Hollenbach, R. E. (1965). *Rev. Sci. Instrum.* **36**, 1617–1620.

[bb112] Basov, D. N. & Chubukov, A. V. (2011). *Nat. Phys.* **7**, 272–276.

[bb71] Battistoni, G., Boehlen, T., Cerutti, F., Chin, P. W., Esposito, L. S., Fassò, A., Ferrari, A., Lechner, A., Empl, A., Mairani, A., Mereghetti, A., Ortega, P. G., Ranft, J., Roesler, S., Sala, P. R., Vlachoudis, V. & Smirnov, G. (2015). *Ann. Nucl. Energy*, **82**, 10–18.

[bb110] Bernhardt, H., Schmitt, A. T., Grabiger, B., Marx-Glowna, B., Loetzsch, R., Wille, H., Bessas, D., Chumakov, A. I., Rüffer, R., Röhlsberger, R., Stöhlker, T., Uschmann, I., Paulus, G. G. & Schulze, K. S. (2020). *Phys. Rev. Res.* **2**, 023365.

[bb70] Bionta, M. R., Lemke, H. T., Cryan, J. P., Glownia, J. M., Bostedt, C., Cammarata, M., Castagna, J. C., Ding, Y., Fritz, D. M., Fry, A. R., Krzywinski, J., Messerschmidt, M., Schorb, S., Swiggers, M. L. & Coffee, R. N. (2011). *Opt. Express*, **19**, 21855–21865. 10.1364/OE.19.021855 22109037

[bb84] Blaj, G., Bhogadi, D., Chang, C., Doering, D., Kenney, C., Kroll, T., Segal, J., Sokaras, D. & Haller, G. (2019). *AIP Conf. Proc.* **2054**, 060037.

[bb82] Blaj, G., Caragiulo, P., Dragone, A., Haller, G., Hasi, J., Kenney, C. J., Kwiatkowski, M., Markovic, B., Segal, J. & Tomada, A. (2016). *Proc. SPIE*, **9968**, 59–68.

[bb49] Boesenberg, U., Samoylova, L., Roth, T., Zhu, D., Terentyev, S., Vannoni, M., Feng, Y., van Driel, T. B., Song, S., Blank, V., Sinn, H., Robert, A. & Madsen, A. (2017). *Opt. Express*, **25**, 2852–2862. 10.1364/OE.25.002852 29519002

[bb62] Brygoo, S., Millot, M., Loubeyre, P., Lazicki, A. E., Hamel, S., Qi, T., Celliers, P. M., Coppari, F., Eggert, J. H., Fratanduono, D. E., Hicks, D. G., Rygg, J. R., Smith, R. F., Swift, D. C., Collins, G. W. & Jeanloz, R. (2015). *J. Appl. Phys.* **118**, 195901.

[bb96] Cerantola, V., McCammon, C., Kupenko, I., Kantor, I., Marini, C., Wilke, M., Ismailova, L., Solopova, N., Chumakov, A., Pascarelli, S. & Dubrovinsky, L. (2015). *Am. Mineral.* **100**, 2670–2681.

[bb5] Cerantola, V., Rosa, A. D., Konôpková, Z., Torchio, R., Brambrink, E., Rack, A., Zastrau, U. & Pascarelli, S. (2021). *J. Phys. Condens. Matter*, **33**, 274003. 10.1088/1361-648X/abfd50 33930892

[bb2] Decking, W. *et al.* (2020). *Nat. Photon.* **14**, 391–397.

[bb80] Descamps, A., Ofori-Okai, K., Appel, V., Cerantola, A., Comley, J., Eggert, L., Fletcher, D., Gericke, S., Göde, S., Humphries, O., Karnbach, A., Lazicki, R., Loetzsch, D., McGonegle, C., Palmer, C., Plueckthun, T., Preston, R., Redmer, D., Senesky, C., Strohm, I., Uschmann, T., White, L., Wollenweber, G., Monaco, J., Wark, J., Hastings, U., Zastrau, G., Gregori, S., Glenzer, S. H. & McBride, E. E. (2020). *Sci. Rep.* **10**, 14564. 10.1038/s41598-020-71350-x PMC7471281 32884061

[bb10] De Vido, M., Ertel, K., Wojtusiak, A., O’Donoghue, N., Tomlinson, S., Divoky, M., Sawicka-Chyla, M., Pilar, J., Mason, P., Phillips, J., Smith, J. M., Banerjee, S., Butcher, T., Edwards, C., Lucianetti, A., Mocek, T. & Collier, J. (2019). *Advanced Solid State Lasers*, p. JTu3A.14. Optical Society of America.

[bb54] Dong, X., Shu, D. & Sinn, H. (2016). *AIP Conf. Proc.* **1741**, 040027.

[bb4] Drake, R. P. (2010). *Phys. Today*, **63**, 28–33.

[bb38] Schneidmiller, E. A. & Yurkov, M. V. (2011). *Photon beam properties at the European XFEL (December (2010). revision)*. Technical Report. Deutsches Elektronen-Synchrotron (DESY), Hamburg Germany.

[bb16] Fiuza, F., Swadling, G. F., Grassi, A., Rinderknecht, H. G., Higginson, D. P., Ryutov, D. D., Bruulsema, C., Drake, R. P., Funk, S., Glenzer, S., Gregori, G., Li, C. K., Pollock, B. B., Remington, B. A., Ross, J. S., Rozmus, W., Sakawa, Y., Spitkovsky, A., Wilks, S. & Park, H. (2020). *Nat. Phys.* **16**, 916–920.

[bb90] Geindre, J. P., Mysyrowicz, A., Santos, A. D., Audebert, P., Rousse, A., Hamoniaux, G., Antonetti, A., Falliès, F. & Gauthier, J. C. (1994). *Opt. Lett.* **19**, 1997. 10.1364/ol.19.001997 19855721

[bb32] Geloni, G., Anton, J., Blank, V., Decking, W., Dong, X., Karabekyan, S., Kearney, S., Kocharyan, V., La Civita, D., Liu, S., *et al.* (2019). *Proceedings of the 39th International Free-Electron Laser Conference (FEL2019)*, 26–30 August 2019, Hamburg, Germany, pp. 242–245. MOP008.

[bb31] Geloni, G., Kocharyan, V. & Saldin, E. (2011). *J. Mod. Opt.* **58**, 1391–1403.

[bb111] Gerber, S., Jang, H., Nojiri, H., Matsuzawa, S., Yasumura, H., Bonn, D. A., Liang, R., Hardy, W. N., Islam, Z., Mehta, A., Song, S., Sikorski, M., Stefanescu, D., Feng, Y., Kivelson, S. A., Devereaux, T. P., Shen, Z. -X., Kao, C. -C., Lee, W. -S., Zhu, D. & Lee, J. S. (2015). *Science*, **350**, 949–952. 10.1126/science.aac6257 26541608

[bb37] Gessler, P., Ali, H., Babies, F., Ballak, K. E., Bamaga, H., Baranasic, B., Bieler, O., Coppola, N., Dornack, K., Eilers, J., *et al.* (2019). *Proceedings of the 17th Biennial International Conference on Accelerator and Large Experimental Physics Control Systems (ICALEPCS2019)*, 5–11 October 2019, New York, NY, USA, pp. 1154–1560. THAPP05.

[bb15] Göde, S., Rödel, K., Zeil, R., Mishra, M., Gauthier, M., Brack, T., Kluge, T., MacDonald, M. J., Metzkes, L., Obst, L., Rehwald, M., Ruyer, C., Schlenvoigt, H. P., Schumaker, W., Sommer, P., Cowan, T. E., Schramm, U., Glenzer, S. & Fiuza, F. (2017). *Phys. Rev. Lett.* **118**, 194801. 10.1103/PhysRevLett.118.194801 28548516

[bb109] Grabiger, B., Marx-Glowna, B., Uschmann, I., Loetzsch, R., Paulus, G. G. & Schulze, K. S. (2020). *Appl. Phys. Lett.* **117**, 201102.

[bb23] Grissonnanche, G., Laliberté, S., Dufour-Beauséjour, M., Matusiak, S., Badoux, S., Tafti, F. F., Michon, A., Riopel, O., Cyr-Choinière, O., Baglo, J. C., Ramshaw, B. J., Liang, R., Bonn, D. A., Hardy, W. N., Krä*mer*, S., LeBoeuf, D., Graf, D., Doiron-Leyraud, N. & Taillefer, L. (2016). *Phys. Rev. B*, **93**, 064513.

[bb47] Grünert, J., Carbonell, M. P., Dietrich, F., Falk, T., Freund, W., Koch, A., Kujala, N., Laksman, J., Liu, J., Maltezopoulos, T., Tiedtke, K., Jastrow, U. F., Sorokin, A., Syresin, E., Grebentsov, A. & Brovko, O. (2019). *J. Synchrotron Rad.* **26**, 1422–1431. 10.1107/S1600577519006611 31490130

[bb92] Hagemann, J., Vassholz, M., Hoeppe, H., Osterhoff, M., Rosselló, J. M., Mettin, R., Seiboth, F., Schropp, A., Möller, J., Hallmann, J., Kim, C., Scholz, M., Boesenberg, U., Schaffer, R., Zozulya, A., Lu, W., Shayduk, R., Madsen, A., Schroer, C. G. & Salditt, T. (2021). *J. Synchrotron Rad.* **28**, 52–63.

[bb77] Hámos, L. von (1934). *Annal. Phys.* **411**, 252–260.

[bb68] Harmand, M., Coffee, R., Bionta, M. R., Chollet, M., French, D., Zhu, D., Fritz, D. M., Lemke, H. T., Medvedev, N., Ziaja, B., Toleikis, S. & Cammarata, M. (2013). *Nat. Photon.* **7**, 215–218.

[bb36] Hauf, S., Heisen, B., Aplin, S., Beg, M., Bergemann, M., Bondar, V., Boukhelef, D., Danilevsky, C., Ehsan, W., Essenov, S., Fabbri, R., Flucke, G., Fulla Marsa, D., Göries, D., Giovanetti, G., Hickin, D., Jarosiewicz, T., Kamil, E., Khakhulin, D., Klimovskaia, A., Kluyver, T., Kirienko, Y., Kuhn, M., Maia, L., Mamchyk, D., Mariani, V., Mekinda, L., Michelat, T., Münnich, A., Padee, A., Parenti, A., Santos, H., Silenzi, A., Teichmann, M., Weger, K., Wiggins, J., Wrona, K., Xu, C., Youngman, C., Zhu, J., Fangohr, H. & Brockhauser, S. (2019). *J. Synchrotron Rad.* **26**, 1448–1461. 10.1107/S1600577519006696 31490132

[bb35] Heisen, B., Boukhelef, D., Esenov, S., Hauf, S., Kozlova, I., Maia, L., Parenti, A., Szuba, J., Weger, K., Wrona, K. & Youngman, C. (2013). *Proceedings of the 14th International Conference on Accelerator and Large Experimental Physics Control Systems (ICALEPCS2013)*, 6–11 October 2013, San Francisco, CA, USA, pp. 1465–1468. FRCOAAB02.

[bb102] Holy, V., Kuběna, I., Ohlidal, I., Lischka, W. & Plotz, W. (1993). *Phys. Rev. B*, **47**, 15896–15903. 10.1103/physrevb.47.15896 10005989

[bb94] Hwang, H., Kim, T., Cynn, H., Vogt, T., Husband, R. J., Appel, K., Baehtz, C., Ball, O. B., Baron, M. A., Briggs, R., Bykov, M., Bykova, E., Cerantola, V., Chantel, J., Coleman, A. L., Dattlebaum, D., Dresselhaus-Marais, L. E., Eggert, J. H., Ehm, L., Evans, W. J., Fiquet, G., Frost, M., Glazyrin, K., Goncharov, A. F., Jenei, Z., Kim, J., Konôpková, Z., Mainberger, J., Makita, M., Marquardt, H., McBride, E. E., McHardy, J. D., Merkel, S., Morard, G., O’Bannon, E. F. III, Otzen, C., Pace, E. J., Pelka, A., Pépin, C. M., Pigott, J. S., Prakapenka, V. B., Prescher, C., Redmer, R., Speziale, S., Spiekermann, G., Strohm, C., Sturtevant, B. T., Velisavljevic, N., Wilke, M., Yoo, C., Zastrau, U., Liermann, H., McMahon, M. I., McWilliams, R. S. & Lee, Y. (2021). *J. Phys. Chem. Lett.* **12**, 3246–3252.

[bb7] Jenei, Z., Liermann, H. P., Husband, R., Méndez, A. S. J., Pennicard, D., Marquardt, H., O’Bannon, E. F., Pakhomova, A., Konopkova, Z., Glazyrin, K., Wendt, M., Wenz, S., McBride, E. E., Morgenroth, W., Winkler, B., Rothkirch, A., Hanfland, M. & Evans, W. J. (2019). *Rev. Sci. Instrum.* **90**, 065114. 10.1063/1.5098993 31255042

[bb107] Karbstein, F. & Mosman, E. A. (2019). *Phys. Rev. D*, **100**, 033002.

[bb60] Kärcher, V., Roling, S., Samoylova, L., Buzmakov, A., Zastrau, U., Appel, K., Yurkov, M., Schneidmiller, E., Siewert, F. & Zacharias, H. (2021). *J. Synchrotron Rad.* **28**, 350–361. 10.1107/S1600577520014563 PMC7842232 33399587

[bb74] Kim, J. B., Göde, S. & Glenzer, S. H. (2016). *Rev. Sci. Instrum.* **87**, 11E328. 10.1063/1.4961089 27910321

[bb67] Kirkwood, H. J., Letrun, R., Tanikawa, T., Liu, J., Nakatsutsumi, M., Emons, M., Jezynski, T., Palmer, G., Lederer, M., Bean, R., Buck, J., Di Dio Cafisio, S., Graceffa, R., Grünert, J., Göde, S., Höppner, H., Kim, Y., Konopkova, Z., Mills, G., Makita, M., Pelka, A., Preston, T. R., Sikorski, M., Takem, C. M. S., Giewekemeyer, K., Chollet, M., Vagovic, P., Chapman, H. N., Mancuso, A. P. & Sato, T. (2019). *Opt. Lett.* **44**, 1650–1653. 10.1364/OL.44.001650 30933113

[bb83] Klačková, I., Blaj, G., Denes, P., Dragone, A., Göde, S., Hauf, S., Januschek, F., Joseph, J. & Kuster, M. (2019). *J. Instrum.* **14**, C01008.

[bb81] Klementiev, K. & Chernikov, R. (2020). kklmn/xrt: Release 1.3.4, https://doi.org/10.5281/zenodo.3838709.

[bb100] Kluge, T., Bussmann, M., Chung, H., Gutt, C., Huang, L. G., Zacharias, M., Schramm, U. & Cowan, T. E. (2016). *Phys. Plasmas*, **23**, 033103.

[bb99] Kluge, T., Gutt, C., Huang, L. G., Metzkes, J., Schramm, U., Bussmann, M. & Cowan, T. E. (2014). *Phys. Plasmas*, **21**, 033110.

[bb46] Koch, A., Risch, J., Freund, W., Maltezopoulos, T., Planas, M. & Grünert, J. (2019). *J. Synchrotron Rad.* **26**, 1489–1495. 10.1107/S1600577519008737 31490136

[bb9] Konôpková, Z., McWilliams, R. S., Gómez-Pérez, N. & Goncharov, A. F. (2016). *Nature*, **534**, 99–101. 10.1038/nature18009 27251283

[bb51] Kujala, N., Freund, W., Liu, J., Koch, A., Falk, T., Planas, M., Dietrich, F., Laksman, J., Maltezopoulos, T., Risch, J., Dall’Antonia, F. & Grünert, J. (2020). *Rev. Sci. Instrum.* **91**, 103101. 10.1063/5.0019935 33138553

[bb41] Lengeler, B., Schroer, C., Tümmler, J., Benner, B., Richwin, M., Snigirev, A., Snigireva, I. & Drakopoulos, M. (1999). *J. Synchrotron Rad.* **6**, 1153–1167.

[bb61] Liermann, H. P., Konôpková, Z., Appel, K., Prescher, C., Schropp, A., Cerantola, V., Husband, R. J., McHardy, J. D., McMahon, M. I., McWilliams, R. S., Pépin, C. M., Mainberger, J., Roeper, M., Berghäuser, A., Damker, H., Talkovski, P., Foese, M., Kujala, N., Ball, O. B., Baron, M. A., Briggs, R., Bykov, M., Bykova, E., Chantel, J., Coleman, A. L., Cynn, H., Dattelbaum, D., Dresselhaus-Marais, L. E., Eggert, J. H., Ehm, L., Evans, W. J., Fiquet, G., Frost, M., Glazyrin, K., Goncharov, A. F., Hwang, H., Jenei, Z., Kim, J.-Y., Langenhorst, F., Lee, Y., Makita, M., Marquardt, H., McBride, E. E., Merkel, S., Morard, G., O’Bannon, E. F., Otzen, C., Pace, E. J., Pelka, A., Pigott, J. S., Prakapenka, V. B., Redmer, R., Sanchez-Valle, C., Schoelmerich, M., Speziale, S., Spiekermann, G., Sturtevant, B. T., Toleikis, S., Velisavljevic, N., Wilke, M., Yoo, C.-S., Baehtz, C., Zastrau, U. & Strohm, C. (2021). *J. Synchrotron Rad.* **28**, 688–706. 10.1107/S1600577521002551 PMC8127375 33949979

[bb95] Lin, J. F., Struzhkin, V. V., Jacobsen, S. D., Hu, M. Y., Chow, P., Kung, J., Liu, H., Mao, H. K. & Hemley, R. J. (2005). *Nature*, **436**, 377–380. 10.1038/nature03825 16034415

[bb25] Liu, J. Y., Hu, J., Zhang, Q., Graf, D., Cao, H. B., Radmanesh, S. M. A., Adams, D. J., Zhu, Y. L., Cheng, G. F., Liu, X., Phelan, W. A., Wei, J., Jaime, M., Balakirev, F., Tennant, D. A., DiTusa, J. F., Chiorescu, I., Spinu, L. & Mao, Z. Q. (2017). *Nat. Mater.* **16**, 905–910. 10.1038/nmat4953 28740190

[bb33] Liu, S., Decking, W., Kocharyan, V., Saldin, E., Serkez, S., Shayduk, R., Sinn, H. & Geloni, G. (2019). *Phys. Rev. Accel. Beams*, **22**, 060704.

[bb63] Loubeyre, P., Brygoo, J., Eggert, J., Celliers, P. M., Spaulding, D. K., Rygg, J. R., Boehly, T. R., Collins, G. W. & Jeanloz, R. (2012). *Phys. Rev. B*, **86**, 144115.

[bb22] Lu, W., Friedrich, B., Noll, T., Zhou, J., Hallmann, G., Ansaldi, T., Roth, T., Serkez, S., Geloni, G., Madsen, A. & Eisebitt, S. (2018). *Rev. Sci. Instrum.* **89**, 063121. 10.1063/1.5027071 29960553

[bb777] Madsen, A., Hallmann, J., Ansaldi, G., Roth, T., Lu, W., Kim, C., Boesenberg, U., Zozulya, A., Möller, J., Shayduk, R., Scholz, M., Bartmann, A., Schmidt, A., Lobato, I., Sukharnikov, K., Reiser, M., Kazarian, K. & Petrov, I. (2021). *J Synchrotron Rad*, **28**, 637–649. 10.1107/S1600577521001302 PMC7941285 33650576

[bb30] Maltezopoulos, T., Dietrich, F., Freund, W., Jastrow, U. F., Koch, A., Laksman, J., Liu, J., Planas, M., Sorokin, A. A., Tiedtke, K. & Grünert, J. (2019). *J. Synchrotron Rad.* **26**, 1045–1051. 10.1107/S1600577519003795 31274426

[bb8] Meza-Galvez, J., Gomez-Perez, N., Marshall, A. S., Coleman, A. L., Appel, K., Liermann, H. P., McMahon, M. I., Konôpková, Z. & McWilliams, R. S. (2020). *J. Appl. Phys.* **127**, 195902.

[bb98] Millot, M., Dubrovinskaia, N., ernok, A., Blaha, S., Dubrovinsky, L., Braun, D. G., Celliers, P. M., Collins, G. W., Eggert, J. H. & Jeanloz, R. (2015). *Science*, **347**, 418–420. 10.1126/science.1261507 25613887

[bb57] Mitzner, R., Siemer, B., Neeb, M., Noll, T., Siewert, F., Roling, S., Rutkowski, M., Sorokin, A. A., Richter, M., Juranic, P., Tiedtke, K., Feldhaus, J., Eberhardt, W. & Zacharias, H. (2008). *Opt. Express*, **16**, 19909–19919. 10.1364/oe.16.019909 19030078

[bb85] Mozzanica, A., Andrä, M., Barten, R., Bergamaschi, A., Chiriotti, S., Brückner, M., Dinapoli, R., Fröjdh, E., Greiffenberg, D., Leonarski, F., Lopez-Cuenca, C., Mezza, D., Redford, S., Ruder, C., Schmitt, B., Shi, X., Thattil, D., Tinti, G., Vetter, S. & Zhang, J. (2018). *Synchrotron Radiat. News*, **31**(6), 16–20.

[bb52] Mozzanica, A., Bergamaschi, A., Dinapoli, R., Graafsma, H., Greiffenberg, D., Henrich, B., Johnson, I., Lohmann, M., Valeria, R., Schmitt, B. & Xintian, S. (2012). *J. Instrum.* **7**, C01019.

[bb103] Müller-Buschbaum, P. (2003). *Anal. Bioanal. Chem.* **376**, 3–10. 10.1007/s00216-003-1869-2 12734612

[bb113] Mydosh, J. A. & Oppeneer, P. M. (2011). *Rev. Mod. Phys.* **83**, 1301–1322.

[bb12] Nakatsutsumi, M., Appel, C., Baehtz, B., Chen, B., Cowan, S., Göde, Z., Konopkova, A., Pelka, G., Priebe, A., Schmidt, K., Sukharnikov, I., Thorpe, Th., Tschentscher, T. & Zastrau, U. (2016). *Plasma Phys. Control. Fusion*, **59**, 014028.

[bb34] Nakatsutsumi, M., Appel, K., Priebe, G., Thorpe, I., Pelka, A., Muller, B. & Tschentscher, Th. (2014). *Scientific Instrument High Energy Density Physics (HED).* Conceptual Design Report: European X-ray Free-Electron Laser Facility GmbH, Schenefeld, Germany.

[bb3] Nakatsutsumi, M. & Tschentscher, Th. (2013). *Scientific Instrument High Energy Density Physics (HED).* Technical Design Report. European X-ray Free-Electron Laser Facility GmbH, Schenefeld, Germany.

[bb75] Obst, L., Göde, S., Rehwald, M., Brack, F., Branco, J., Bock, S., Bussmann, M., Cowan, T. E., Curry, C. B., Fiuza, F., Gauthier, M., Gebhardt, R., Helbig, U., Huebl, A., Hübner, U., Irman, A., Kazak, L., Kim, J. B., Kluge, T., Kraft, S., Loeser, M., Metzkes, J., Mishra, R., Rödel, C., Schlenvoigt, H., Siebold, M., Tiggesbäumker, J., Wolter, S., Ziegler, T., Schramm, U., Glenzer, S. H. & Zeil, K. (2017). *Sci. Rep.* **7**, 10248.

[bb66] Palmer, G., Kellert, M., Wang, J., Emons, M., Wegner, U., Kane, D., Pallas, F., Jezynski, T., Venkatesan, S., Rompotis, D., Brambrink, E., Monoszlai, B., Jiang, M., Meier, J., Kruse, K., Pergament, M. & Lederer, M. J. (2019). *J. Synchrotron Rad.* **26**, 328–332. 10.1107/S160057751900095X 30855239

[bb65] Phillips, J. P., Banerjee, S., Mason, P., Smith, J., Spear, J., De Vido, M., Ertel, K., Butcher, T., Quinn, G., Clarke, D., Edwards, C., Hernandez-Gomez, C. & Collier, J. (2021). *Opt. Lett.* **46**, 1808. 10.1364/OL.419861 33857075

[bb64] Phillips, P. J., Mason, P., Ertel, K., Smith, J., De-Vido, M., Butcher, T., Tomlinson, S., Suarez-Merchan, J. E., Lintern, A., Costello, B., Hollingham, I., Norton, A., Tyldesley, M., Hernandez-Gomez, C., Edwards, C., Collier, J., Höppner, H., Toncian, T., Zastrau, U. & Möller, D. (2019). *Proc. SPIE*, **10898**, 108980K.

[bb43] Pikuz, T., Faenov, A., Matsuoka, T., Matsuyama, S., Yamauchi, K., Ozaki, N., Albertazzi, B., Inubushi, Y., Yabashi, M., Tono, K., Sato, Y., Yumoto, H., Ohashi, H., Pikuz, S., Grum-Grzhimailo, A. N., Nishikino, M., Kawachi, T., Ishikawa, T. & Kodama, R. (2015). *Sci. Rep.* **5**, 17713. 10.1038/srep17713 PMC4669527 26634431

[bb778] Prencipe, I., Fuchs, J., Pascarelli, S., Schumacher, D. W., Stephens, R. B., Alexander, N. B., Briggs, R., Büscher, M., Cernaianu, M. O., Choukourov, A., De Marco, M., Erbe, A., Fassbender, J., Fiquet, G., Fitzsimmons, P., Gheorghiu, C., Hund, J., Huang, L. G., Harmand, M., Hartley, N. J., Irman, A., Kluge, T., Konopkova, Z., Kraft, S., Kraus, D., Leca, V., Margarone, D., Metzkes, J., Nagai, K., Nazarov, W., Lutoslawski, P., Papp, D., Passoni, M., Pelka, A., Perin, J. P., Schulz, J., Smid, M., Spindloe, C., Steinke, S., Torchio, R., Vass, C., Wiste, T., Zaffino, R., Zeil, K., Tschentscher, T., Schramm, U. & Cowan, T. E. (2017). *High Pow Laser Sci Eng*, **5**, e17.

[bb76] Preston, T. R., Göde, S., Schwinkendorf, J., Appel, K., Brambrink, E., Cerantola, V., Höppner, H., Makita, M., Pelka, A., Prescher, C., Sukharnikov, K., Schmidt, A., Thorpe, I., Toncian, T., Amouretti, A., Chekrygina, D., Falcone, R. W., Falk, K., Fletcher, L. B., Galtier, E., Harmand, M., Hartley, N. J., Hau-Riege, S. P., Heimann, P., Huang, L. G., Humphries, O. S., Karnbach, O., Kraus, D., Lee, H. J., Nagler, B., Ren, S., Schuster, A. K., Smid, M., Voigt, K., Zhang, M. & Zastrau, U. (2020). *J. Instrum.* **15**, P11033.

[bb105] Randolph, L., Banjafar, M., Preston, T. R., Yabuuchi, T., Makita, M., Dover, N. P., Rödel, C., Göde, S., Inubushi, Y., Jakob, G., Kaa, J.,. Kon, A., Koga, J. K., Ksenzov, D., Matsuoka, T., Nishiuchi, M., Paulus, M., Schon, F., Sueda, K., Sentoku, Y., Togashi, T., Vafaee-Khanjani, M., Bussmann, M., Cowan, T. E., Kläui, M., Fortmann-Grote, C., Mancuso, A. P., Kluge, T., Gutt, C. & Nakatsutsumi, M. (2020). *arXiv*: 2012.15076 [physics. plasm-ph].

[bb87] Redford, S., Andrä, M., Barten, R., Bergamaschi, A., Brückner, M., Chiriotti, S., Dinapoli, R., Fröjdh, E., Greiffenberg, D., Kim, K. S., Lee, J. H., Lopez-Cuenca, C., Meyer, M., Mezza, D., Mozzanica, A., Park, S., Ruder, C., Schmitt, B., Shi, X., Thattil, D., Tinti, G., Vetter, S. & Zhang, J. (2020). *J. Instrum.* **15**, C02025.

[bb86] Redford, S., Andrä, M., Barten, R., Bergamaschi, A., Brückner, M., Dinapoli, R., Fröjdh, E., Greiffenberg, D., Lopez-Cuenca, C., Mezza, D., Mozzanica, A., Ramilli, M., Ruat, M., Ruder, C., Schmitt, B., Shi, X., Thattil, D., Tinti, G., Vetter, S. & Zhang, J. (2018). *J. Instrum.* **13**, C01027.

[bb69] Riedel, R., Al-Shemmary, A., Gensch, M., Golz, T., Harmand, M., Medvedev, N., Prandolini, M. J., Sokolowski-Tinten, K., Toleikis, S., Wegner, U., Ziaja, B., Stojanovic, N. & Tavella, F. (2013). *Nat. Commun.* **4**, 1731. 10.1038/ncomms2754 23591898

[bb59] Roling, S., Appel, K., Braun, S., Buzmakov, A., Chubar, O., Gawlitza, P., Samoylova, L., Siemer, B., Schneidmiller, E., Sinn, H., Siewert, F., Tschentscher, T., Wahlert, F., Wöstmann, M., Yurkov, M. & Zacharias, H. (2017). *Proc. SPIE*, **9210**, 92100B.

[bb104] Roth, S. V. (2016). *J. Phys. Condens. Matter*, **28**, 403003. 10.1088/0953-8984/28/40/403003 27537198

[bb14] Ruyer, C., Bolaños, S., Albertazzi, B., Chen, S. N., Antici, P., Böker, J., Dervieux, V., Lancia, L., Nakatsutsumi, M., Romagnani, L., Shepherd, R., Swantusch, M., Borghesi, M., Willi, O., Pépin, H., Starodubtsev, M., Grech, M., Riconda, C., Gremillet, L. & Fuchs, J. (2020). *Nat. Phys.* **16**, 983–988.

[bb50] Samoylova, L., Boesenberg, U., Chumakov, A., Kaganer, V., Petrov, I., Roth, T., Rüffer, R., Sinn, H., Terentyev, S. & Madsen, A. (2019). *J. Synchrotron Rad.* **26**, 1069–1072. 10.1107/S1600577519004880 31274429

[bb108] Schlenvoigt, H., Heinzl, T., Schramm, U., Cowan, T. E. & Sauerbrey, R. (2016). *Phys. Scr.* **91**, 023010.

[bb72] Schmidt, A. & Dommach, M. (2015). *HV Guidelines for Scientific Instruments.* Technical Report. European X-ray Free-Electron Laser Facility GmbH, Schenefeld, Germany.

[bb28] Scholz, M. & Zhao, Z. T. (2019). *Proceedings of the 39th International Free-Electron Laser Conference (FEL2019)*, 26–30 August 2019, Hamburg, Germany. MOA04.

[bb45] Schropp, A., Hoppe, R., Meier, V., Patommel, J., Seiboth, F., Lee, H. J., Nagler, B., Galtier, E. C., Arnold, B., Zastrau, U., Hastings, J. B., Nilsson, D., Uhlén, F., Vogt, U., Hertz, H. M. & Schroer, C. G. (2013). *Sci. Rep.* **3**, 1633. 10.1038/srep01633 PMC3620670 23567281

[bb91] Schropp, A., Hoppe, R., Meier, V., Patommel, J., Seiboth, F., Ping, Y., Hicks, D. G., Beckwith, M. A., Collins, G. W., Higginbotham, A., Wark, J. S., Lee, H. J., Nagler, B., Galtier, E. C., Arnold, B., Zastrau, U., Hastings, J. B. & Schroer, C. G. (2015). *Sci. Rep.* **5**, 11089. 10.1038/srep11089 PMC4650669 26086176

[bb44] Schropp, A., Patommel, J., Seiboth, F., Arnold, B., Galtier, E. C., Lee, H. J., Nagler, B., Hastings, J. B. & Schroer, C. G. (2012). *Proc. SPIE*, **8504**, 73–79.

[bb39] Sinn, H., Dommach, M., Dickert, B., Di Felice, M., Dong, X., Eidam, J., Finze, D., Freijo-Martin, I., Gerasimova, N., Kohlstrunk, N., La Civita, D., Meyn, F., Music, V., Neumann, M., Petrich, M., Rio, B., Samoylova, L., Schmidtchen, S., Störmer, M., Trapp, A., Vannoni, M., Villanueva, R. & Yang, F. (2019). *J. Synchrotron Rad.* **26**, 692–699. 10.1107/S1600577519003461 31074432

[bb101] Šmíd, M., Baehtz, A., Pelka, A., Laso García, S., Göde, J., Grenzer, T., Kluge, Z., Konopkova, M., Makita, I., Prencipe, I., Preston, T. R., Rödel, M. & Cowan, T. E. (2020). *Rev. Sci. Instrum.* **91**, 123501. 10.1063/5.0021691 33379989

[bb53] Sorokin, A. A., Bican, Y., Bonfigt, S., Brachmanski, M., Braune, M., Jastrow, U. F., Gottwald, A., Kaser, H., Richter, M. & Tiedtke, K. (2019). *J. Synchrotron Rad.* **26**, 1092–1100. 10.1107/S1600577519005174 PMC6613123 31274432

[bb21] Sperling, P., Gamboa, E. J., Lee, H. J., Chung, H. K., Galtier, Y., Omarbakiyeva, Y., Reinholz, H., Röpke, U., Zastrau, J., Hastings, J., Fletcher, L. B. & Glenzer, S. H. (2015). *Phys. Rev. Lett.* **115**, 115001. 10.1103/PhysRevLett.115.115001 26406836

[bb93] Spiekermann, G., Kupenko, I., Petitgirard, S., Harder, M., Nyrow, A., Weis, C., Albers, C., Biedermann, N., Libon, L., Sahle, C. J., Cerantola, V., Glazyrin, K., Konôpková, Z., Sinmyo, R., Morgenroth, W., Sergueev, I., Yavaş, H., Dubrovinsky, L., Tolan, M., Sternemann, C. & Wilke, M. (2020). *J. Synchrotron Rad.* **27**, 414–424. 10.1107/S1600577519017041 PMC7064108 32153280

[bb1] Tschentscher, T., Bressler, C., Grünert, J., Madsen, A., Mancuso, A., Meyer, M., Scherz, A., Sinn, H. & Zastrau, U. (2017). *Appl. Sci.* **7**, 592.

[bb114] Ueda, K., Oh, T., Yang, B. J., Kaneko, R., Fujioka, J., Nagaosa, N. & Tokura, Y. (2017). *Nat. Commun.* **8**, 15515. 10.1038/ncomms15515 PMC5458080 28537276

[bb97] Umemoto, K., Wentzcovitch, R. M. & Allen, P. B. (2006). *Science*, **311**, 983–986. 10.1126/science.1120865 16484489

[bb40] Vannoni, M., Freijo Martín, I., Schmidtchen, S., Baumann, T. M., Meyer, M. & Music, V. (2019). *J. Synchrotron Rad.* **26**, 1110–1114. 10.1107/S1600577519005381 PMC6613111 31274434

[bb20] Vinko, S. M., Ciricosta, O., Cho, B. I., Engelhorn, K., Chung, H., Brown, C. R. D., Burian, J., Chalupský, J., Falcone, R. W., Graves, C., Hájková, V., Higginbotham, A., Juha, L., Krzywinski, J., Lee, H. J., Messerschmidt, M., Murphy, C. D., Ping, Y., Scherz, A., Schlotter, W., Toleikis, S., Turner, J. J., Vysin, L., Wang, T., Wu, B., Zastrau, U., Zhu, D., Lee, R. W., Heimann, P. A., Nagler, B. & Wark, J. S. (2012). *Nature*, **482**, 59–62. 10.1038/nature10746 22278059

[bb13] Wang, T., Toncian, T., Wei, M. S. & Arefiev, A. V. (2019). *Phys. Plasmas*, **26**, 013105.

[bb26] Weise, H. & Decking, W. (2017). *Proceedings of the International Free Electron Laser Conference (FEL’17)*, 20–25 August 2017, Santa Fe, NM, USA, pp. 9–13. MOC03.

[bb19] Wheeler, J. A., Borot, A., Monchocé, S., Vincenti, H., Ricci, A., Malvache, A., Lopez-Martens, R. & Quéré, F. (2012). *Nat. Photon.* **6**, 829–833.

[bb17] Wilks, S. C., Langdon, A. B., Cowan, T. E., Roth, M., Singh, M., Hatchett, S., Key, M. H., Pennington, D., MacKinnon, A. & Snavely, R. A. (2001). *Phys. Plasmas*, **8**, 542–549.

[bb55] Wollenweber, L., Preston, T. R., Descamps, A., Cerantola, V., Comley, A., Eggert, J. H., Fletcher, L. B., Geloni, G., Gericke, D. O., Glenzer, S. H., Göde, S., Hastings, J., Humphries, O. S., Jenei, A., Karnbach, O., Konopkova, Z., Loetzsch, R., Marx-Glowna, B., McBride, E. E., McGonegle, D., Monaco, G., Ofori-Okai, B. K., Palmer, C. A. J., Plückthun, C., Redmer, R., Strohm, C., Thorpe, I., Tschentscher, T., Uschmann, I., Wark, J. S., White, T. G., Appel, K., Gregori, G. & Zastrau, U. (2021). *Rev. Sci. Instrum.* **92**, 013101. 10.1063/5.0022886 33514249

[bb24] Wosnitza, J., Zvyagin, S. A. & Zherlitsyn, S. (2016). *Rep. Prog. Phys.* **79**, 074504. 10.1088/0034-4885/79/7/074504 27310818

[bb58] Wöstmann, M., Mitzner, T., Noll, S., Roling, B., Siemer, F., Siewert, S., Eppenhoff, F., Wahlert, F. & Zacharias, H. (2013). *J. Phys. B At. Mol. Opt. Phys.* **46**, 164005.

[bb56] Yoneda, H., Inubushi, Y., Nagamine, K., Michine, Y., Ohashi, H., Yumoto, H., Yamauchi, K., Mimura, H., Kitamura, H., Katayama, T., Ishikawa, T. & Yabashi, M. (2015). *Nature*, **524**, 446–449. 10.1038/nature14894 26310765

[bb18] Zastrau, U., Audebert, P., Bernshtam, V., Brambrink, E., Kämpfer, T., Kroupp, E., Loetzsch, R., Maron, Y., Ralchenko, Y., Reinholz, H., Röpke, G., Sengebusch, A., Stambulchik, E., Uschmann, I., Weingarten, L. & Förster, E. (2010). *Phys. Rev. E*, **81**, 026406. 10.1103/PhysRevE.81.026406 20365664

[bb79] Zastrau, U., Brown, C. R. D., Döppner, T., Glenzer, S. H., Gregori, G., Lee, H. J., Marschner, H., Toleikis, S., Wehrhan, O. & Förster, E. (2012). *J. Instrum.* **7**, P09015.

[bb78] Zastrau, U., Woldegeorgis, A., Förster, E., Loetzsch, R., Marschner, H. & Uschmann, I. (2013). *J. Instrum.* **8**, P10006.

[bb11] Zastrau, U., McMahon, M., Appel, K., Baehtz, C., Brambrink, E., Briggs, R., Butcher, T., Cauble, B., Chen, B. & Damker, H. (2017). *Dynamic Laser Compression Experiments at the HED Instrument of European XFEL.* Conceptual Design Report. European X-ray Free-Electron Laser Facility GmbH, Schenefeld, Germany.

[bb48] Zhu, D., Cammarata, M., Feldkamp, J. M., Fritz, D. M., Hastings, J. B., Lee, S., Lemke, H. T., Robert, A., Turner, J. L. & Feng, Y. (2012). *Appl. Phys. Lett.* **101**, 034103.

[bb42] Zozulya, A., Batchelor, L., Appel, K., Boesenberg, U., Hallmann, J., Kim, C., Lobato, I., Lu, W., Mammen, C., Möller, J., Roth, T., Samoylova, L., Scholz, M., Shayduk, R., Sukharnikov, K. & Madsen, A. (2019). *Proc. SPIE*, **11111**, 111110H.

